# Supplementary material for: A reference map of murine cardiac transcription factor chromatin occupancy identifies dynamic and conserved enhancers
Source: Nat Commun. 2019 Oct 28;10:4907. doi: 10.1038/s41467-019-12812-3 (PMC6817842; doi:10.1038/s41467-019-12812-3)
Supplement: Supplementary file 2 — Supplementary Information [file 41467_2019_12812_MOESM2_ESM.pdf]

SUPPLEMENTARY INFORMATION

Akerberg, Gu et al., “A reference map of murine cardiac transcription factor chromatin occupancy identifies dynamic and conserved enhancers”

Supplementary Fig. 1

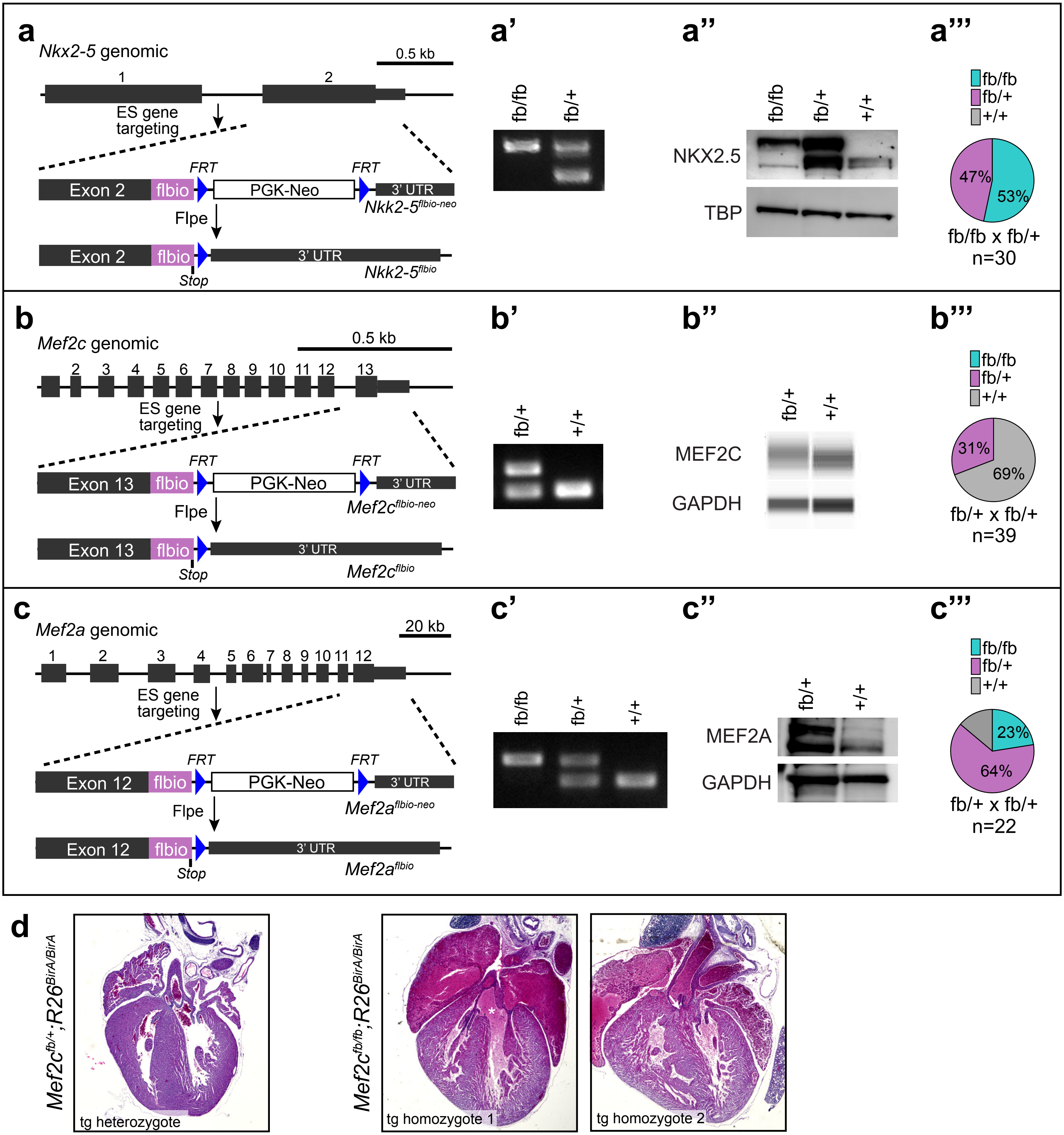

Supplementary Figure 1. Epitope-tagged knockin alleles of Nkx2-5, Mef2c, and Mef2a for in vivo TF biotinylation. A sequence encoding FLAG and BIO peptides was knocked into the Nkx2-5, Mef2c, and Mef2a locus just before the stop codon (**a-c**). The Frt-flanked Neo resistance cassette was removed in the germline by Flp recombinase. **a'-c'**. PCR genotyping of mice using primers that flank the flbio-encoding sequence. **a''-c''**. Western blot of heart extracts probed with NKX2-5, MEF2C, or MEF2A antibodies, respectively. TBP or GAPDH was used as a loading control. **a'''-c'''**. Summary of frequency of progeny obtained from the indicated crosses at weaning. Mef2c$^{fb/fb}$ mice died perinatally. Nkx2-5$^{fb/fb}$ and Mef2a$^{fb/fb}$ mice survived normally. **d.** Neonatal Mef2c$^{fb/fb}$ homozygotes died perinatally with ventricular septal defects (*) and overriding aorta (arrow). Source data for panels a'',b'',c'' are provided in SourceData_Supplementary.

Supplementary Fig. 2

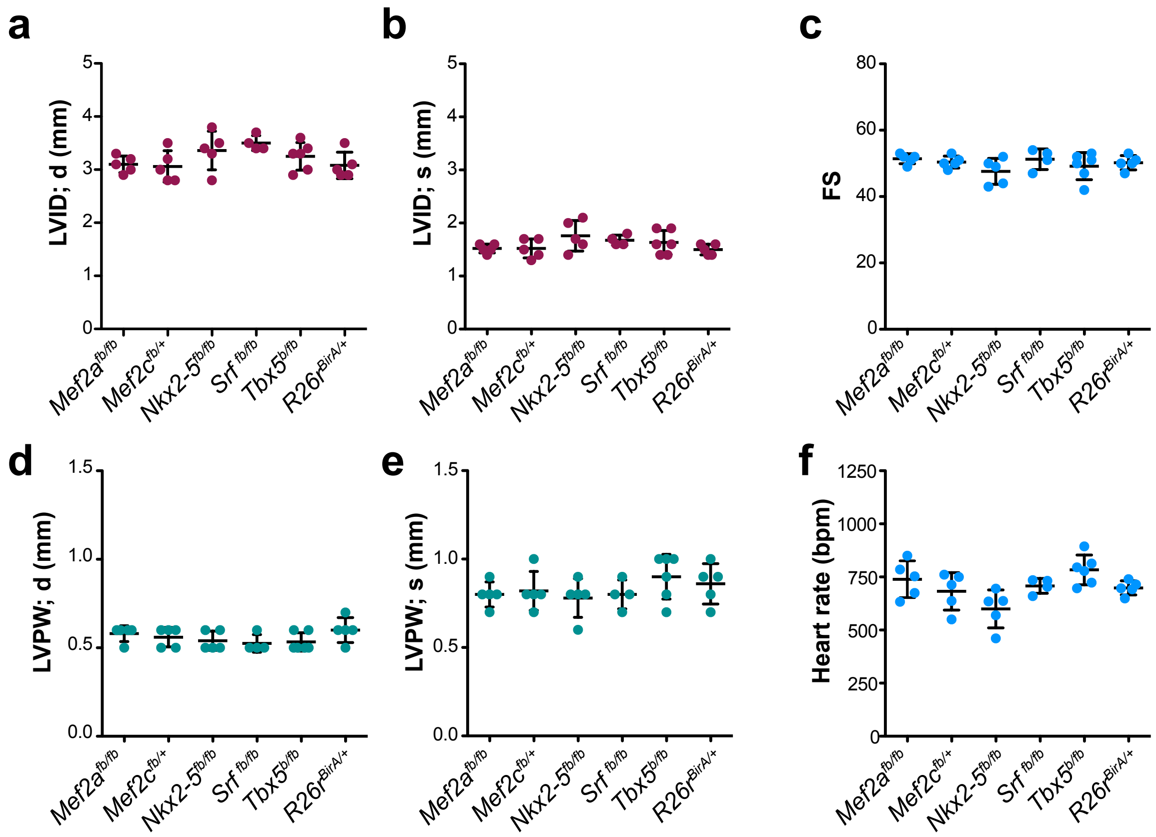

Supplementary Figure 2. Echocardiograms demonstrate normal heart function of BIO-tagged knock-in mice. Adult Mef2a$^{fb/fb}$, Mef2cfb/+, Nkx2-5$^{fb/fb}$, Srf$^{fb/fb}$, Tbx5$^{fb/fb}$, and Rosa26$^{BirA/+}$ (control) mice underwent echocardiography. These mice had normal heart function based on measurements of LV internal diameter in diastole (LVID;d) and systole (LVID;s), fractional shortening (FS%), left ventricular posterior wall thickness in diastolc (LVPW;d) and systol (LVPW;s), and heart rate. Each point in each graph represents a separate mouse, ages P42-P48. Box indicates median and 25th and 75th quartiles. Whiskers represent s.d. Source data for panels a-f are provided in SourceData_Supplementary.

Supplementary Fig. 3

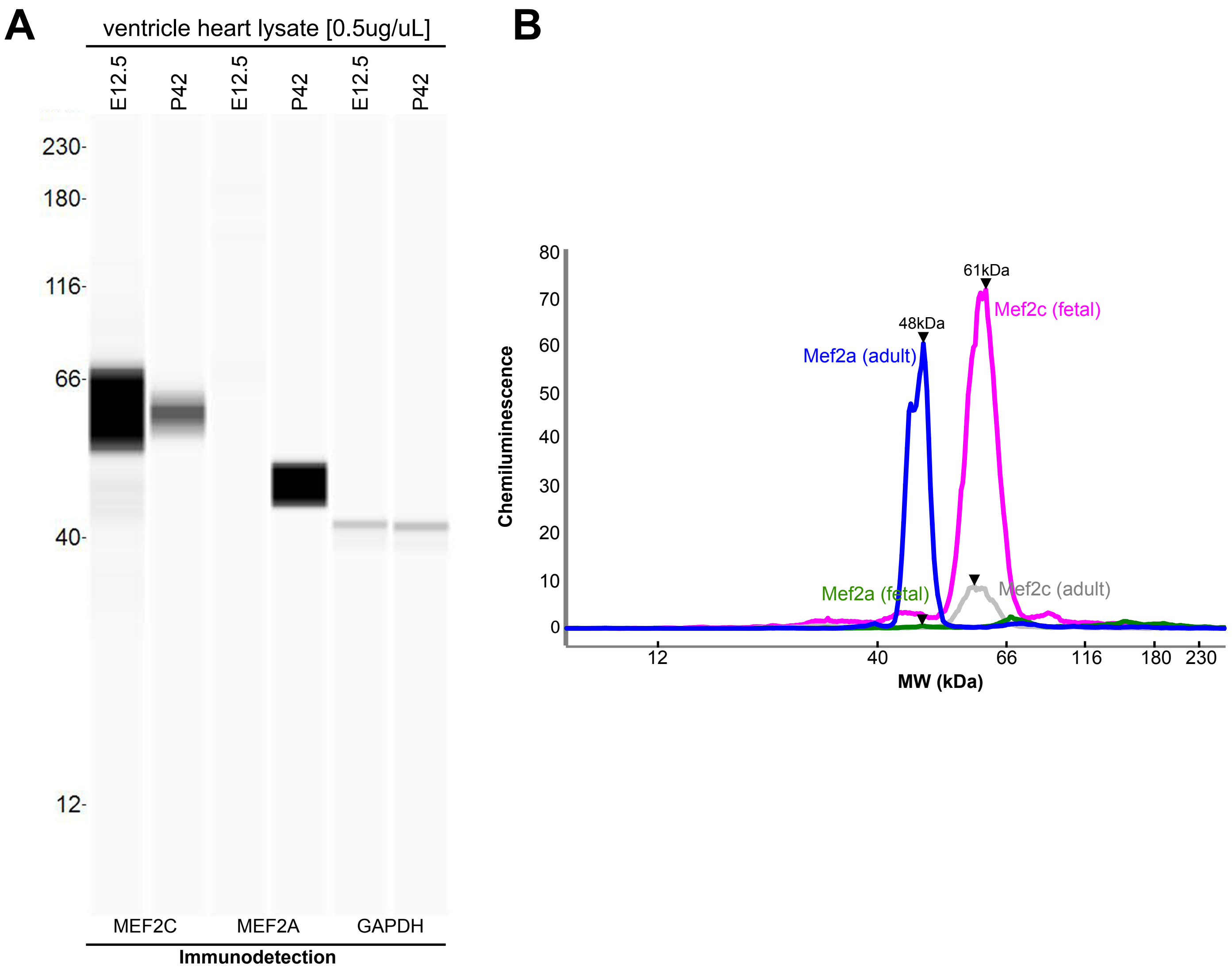

Supplementary Figure 3. MEF2A and MEF2C protein expression in fetal and adult hearts. Heart protein extracts prepared at E12.5 and P42 were analyzed using a capillary western instrument and antibodies specific to MEF2A or MEF2C. MEF2C protein was much more abundant in fetal compared to adult heart, whereas MEF2A protein was more abundant in adult compared to fetal heart. GAPDH was used as an internal loading control.

Supplementary Fig. 4

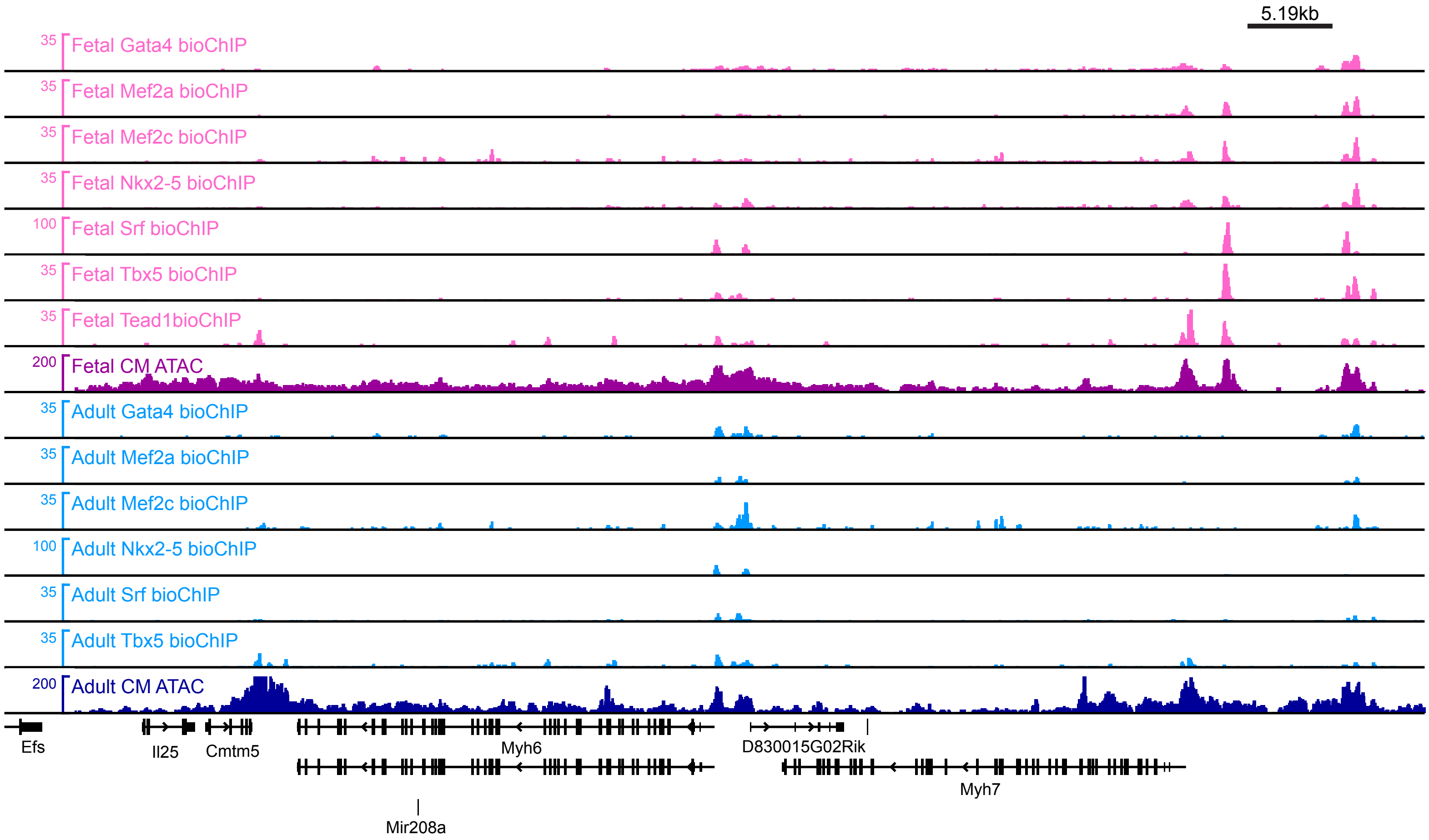

Supplementary Figure 4. Dynamic TF binding regulates stage-specific gene expression. IGV snapshot of dynamic fetal and adult TF binding at *Myh6* and *Myh7* genes. Myosin heavy chain alpha (*Myh6*) is highly expressed in the adult heart and adult bioChIP-seq revealed greater ChIP-seq signal upstream of *Myh6* for nearly all TFs compared to fetal. Conversely, myosin heavy chain beta (*Myh7*) is more highly expressed during heart development (fetal) and bioChIP-seq indicated fetal-specific TF enrichment upstream of *Myh7*. “CM ATAC”, cardiomyocyte-specific ATAC-sequencing.

Supplementary Fig. 5

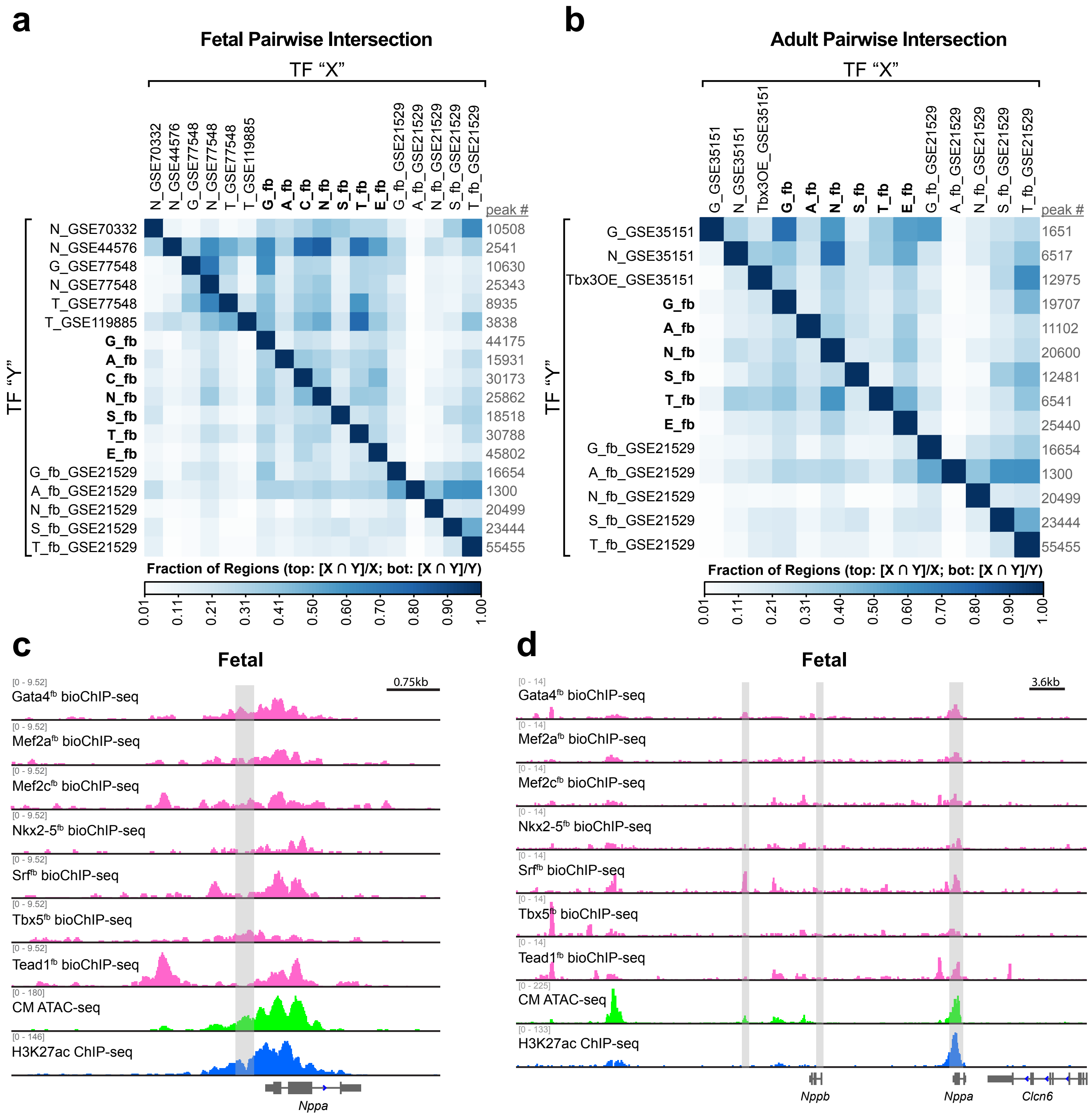

Supplementary Figure 5: Comparison of bioChIP-seq data to previously published cardiac ChIP-seq data. a-b. Heatmaps show pairwise intersection between indicated datasets. Values above the diagonal use the samples along the top as the denominator for calculating fractional overlap, whereas samples below the diagonal use the samples along the right as the denominator. **“**_fb” indicates bioChIP-seq, as opposed to antibody-mediated ChIP-seq. GSE21529, epitope tagged TF expression in HL1 cells. GSE70332, E12.5 pulmonary veins, atria, and part of ventricles. GSE44576, E11.5 mouse heart. GSE77548, mouse ESC-derived CMs, ChIP-exo. GSE119855, E9.5 mouse heart. GSE35151, adult heart with TBX3 overexpression in CMs. **c-d.** IGV browser snapshots with fetal bioChIP-seq, cardiomyocyte (CM)-specific ATAC-seq and E11.5 H3K27ac ChIP-seq datas. BioChIP-seq data from fetal hearts recapitulates similar GATA4, NKX2-5, and TBX5 occupancies (gray bars) at the *Nppa* promoter and *Nppa*-*Nppb* gene cluster as previously described by Luna-Zurita, et al., and Ang, et al., respectively. Source data for panels a-b are provided in SourceData_Supplementary.

Supplementary Fig. 6

a

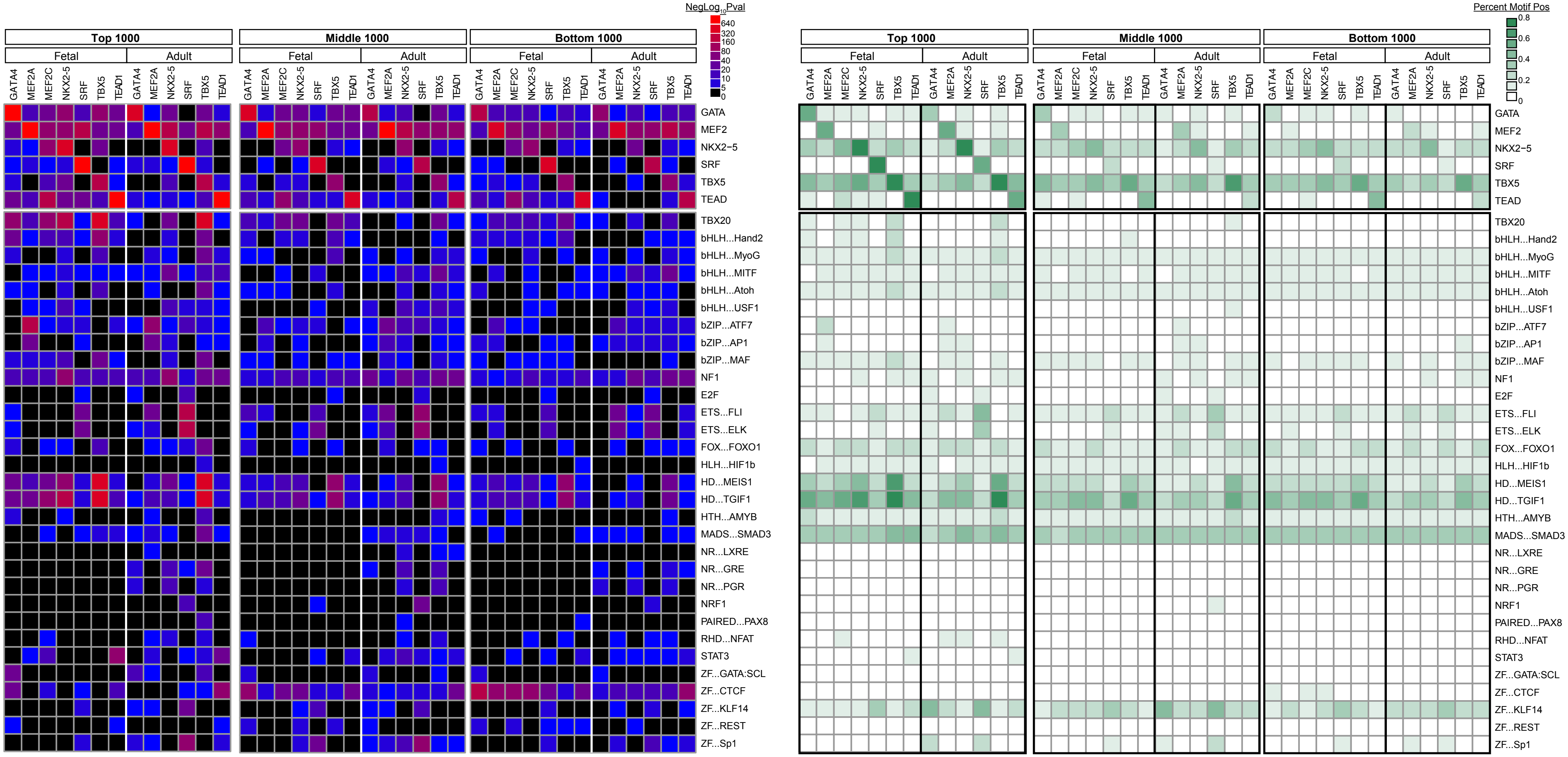

Supplementary Figure 6. Motif enrichment analysis. a. Motif enrichment compared to random background matched for GC content in the top, middle and bottom 1000 peaks from each TF BioChIP-seq sample, ranked by ChIP signal. The analysis of the top 1000 peaks is also shown from Fig. 1. Motif enrichment in the top, middle, and bottom 1000 peaks (summit ± 100 bp) was evaluated using Homer. All non-redundant motifs with an adjusted negative log P-value ≥5 are displayed. The middle and bottom 1000 peaks showed a similar overall pattern to the top 1000 peaks, suggesting that TF interactions do not differ markedly as a function of binding strength. Source data for panel a is provided in SourceData_Supplementary.

b

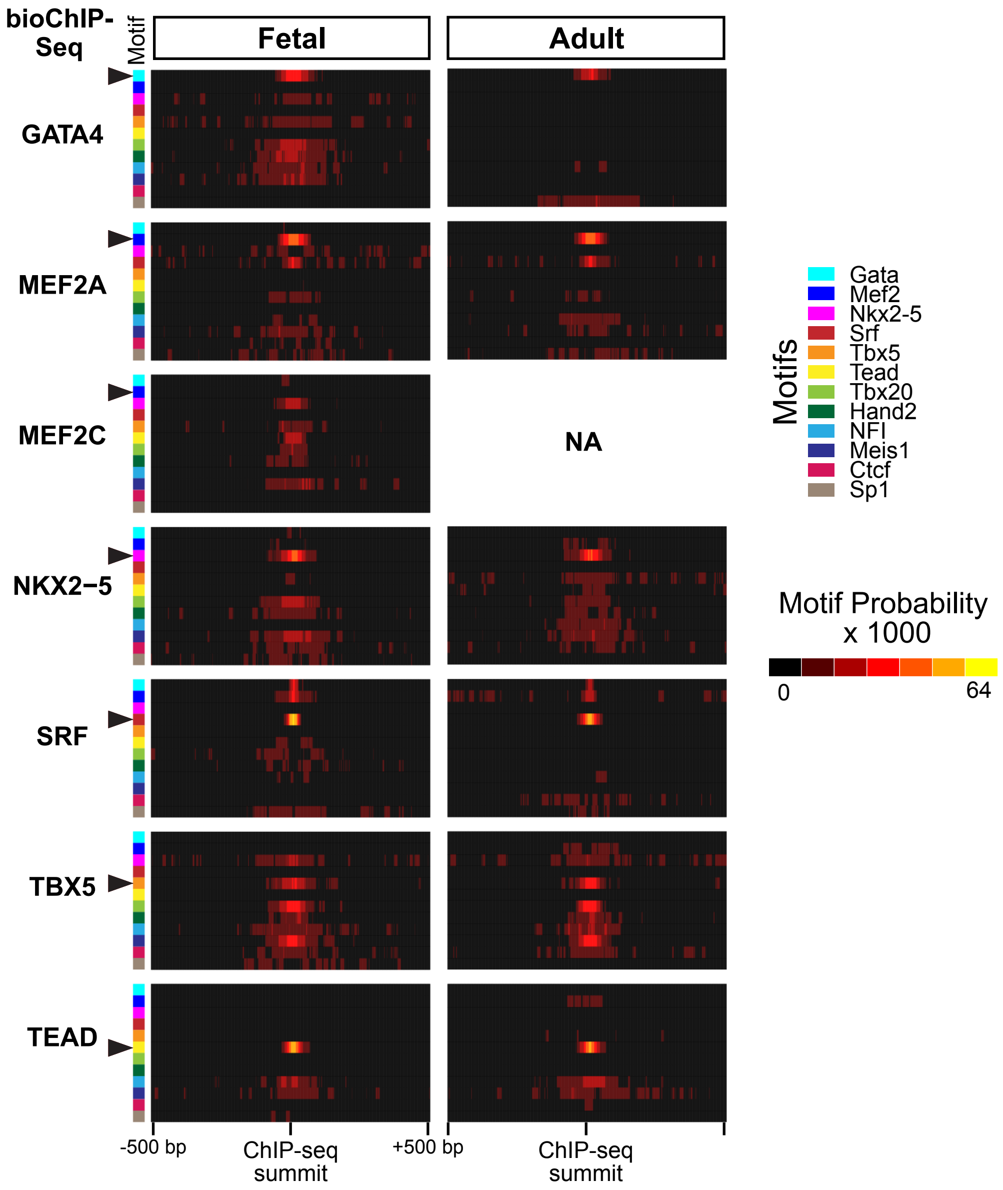

Supplementary Figure 6, continued. Motif enrichment analysis. b. Heatmap of the enrichment (motif probability) of the indicated motifs (rows marked by colored squares) at the center of the top 1000 bioChIP-seq regions, compared to the edges. The colored boxes along the left edge of each row identifies the motif analyzed in that row.

Supplementary Fig. 7

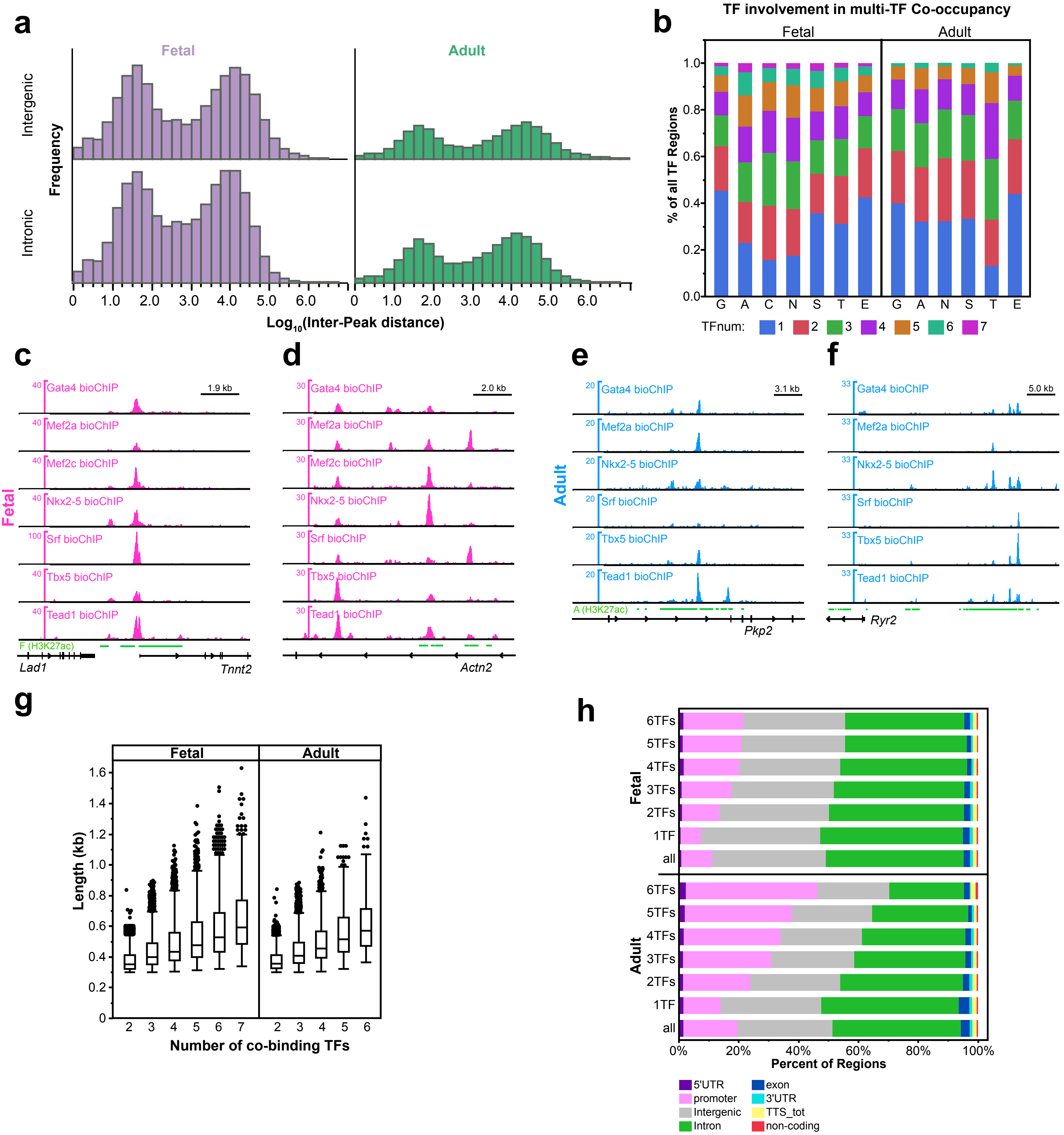

Supplementary Figure 7. Collaborative TF co-binding. a. Histogram of distance between adjacent bioChIP-seq peak centers in intronic or intergenic regions, at Fetal and Adult stages. **b.** TF involvement in multi-TF co-occupancy. The stacked bar plot shows the fraction of a TF's peaks that fall within regions co-occupied by 1-7 different TFs. **c-f.** Genome browser snapshots of genomic regions that are co-occupied by multiple cardiac TFs in fetal but not adult heart (a-b), or adult but not fetal heart (c-d). **g.** Length of regions co-occupied by two more more TFs. Co-occupied regions were defined as regions in which the distance between consecutive TF peak centers was less that 300 bp. The box plot indicates median and 1st and 3rd quartiles. Whiskers indicate 1.5 times the interquartile range. **h.** Location of regions co-bound by multiple TFs with respect to genome annotations. The fraction of regions within promoters (TSS ± 2 kb) increased as the number of co-binding TFs increased, especially in adult heart. Source data for panels a-b,g,h are provided in SourceData_Supplementary.

Supplementary Fig. 8

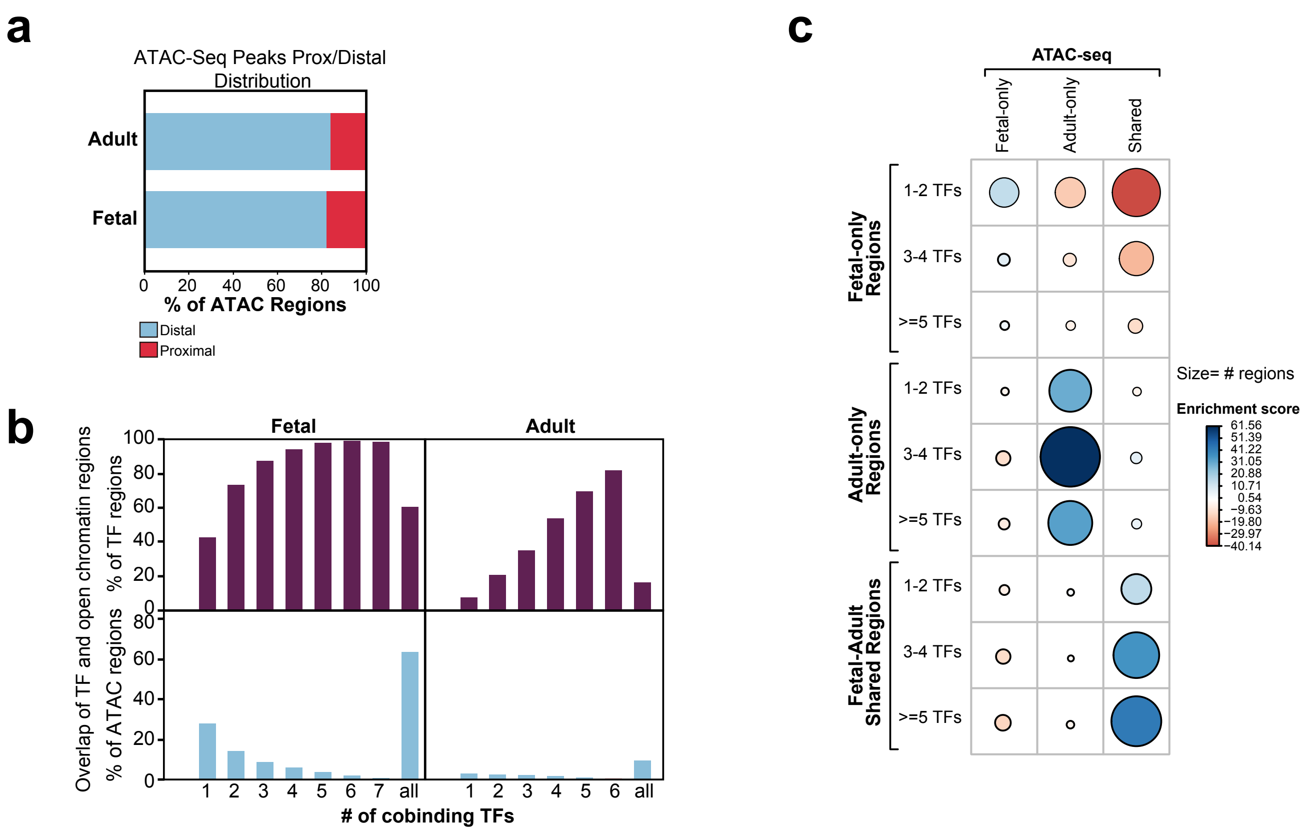

Supplementary Figure 8. Accessible chromatin and TF cobound regions.
a. Accessible chromatin distribution between proximal (TSS ± 2 kb) and distal regions in fetal and adult heart, as assessed by ATAC-seq. **b.** TF regions with indicated number of co-binding TFs that intersected ATAC regions are plotted as a fraction of TF regions or as a fraction of ATAC regions. Most regions occupied by multiple TFs overlapped ATAC regions, especially in fetal heart. **c.** Stage-specific TF co-occupancy was significantly associated with stage-specific chromatin accessibility. Bubble plot shows Chi-square test enrichment score (color) and number of regions in each intersection (circle size). Source data for panels a-c are provided in SourceData_Supplementary.

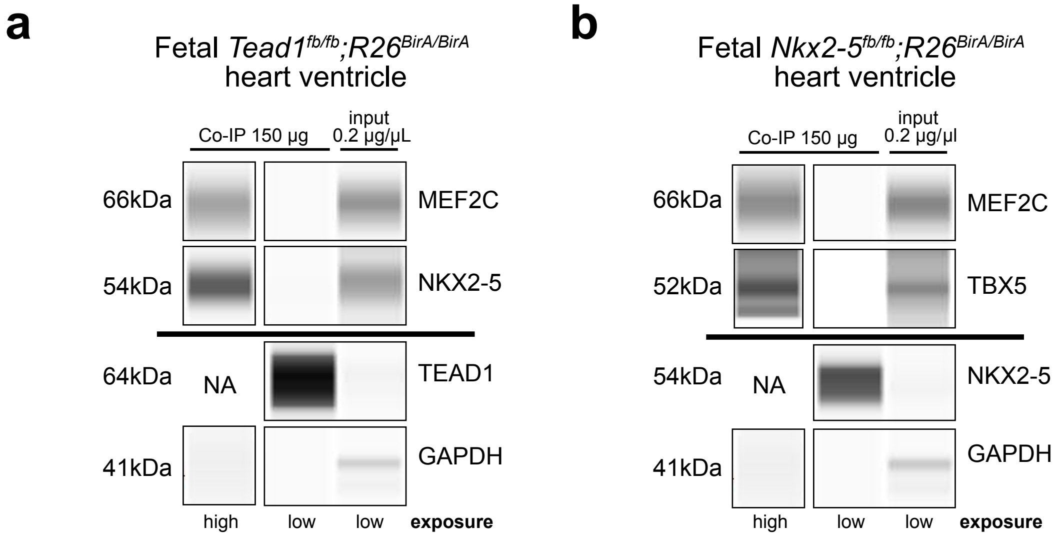

Supplementary Figure 9. Interactions between cardiac TFs in fetal heart. Biotinylated TEAD1^{fb} (a) or NKX2-5^{fb} (b) were pulled down from fetal ventricle protein extracts using streptavidin beads. Co-precipitated proteins were analyzed by capillary western. The precipitated protein sample is shown at low and high exposures to better display both the biotinylated protein and the co-precipitated proteins. **a.** TEAD1^{fb} protein co-precipitated MEF2C and NKX2-5. The negative control, GAPDH, was not enriched in the precipitated protein sample. **b.** NKX2-5^{fb} protein co-precipitated MEF2C and TBX5. The negative control, GAPDH, was not enriched in the precipitated protein sample. Source data for panels a-b are provided in SourceData_images.

Supplementary Fig. 10

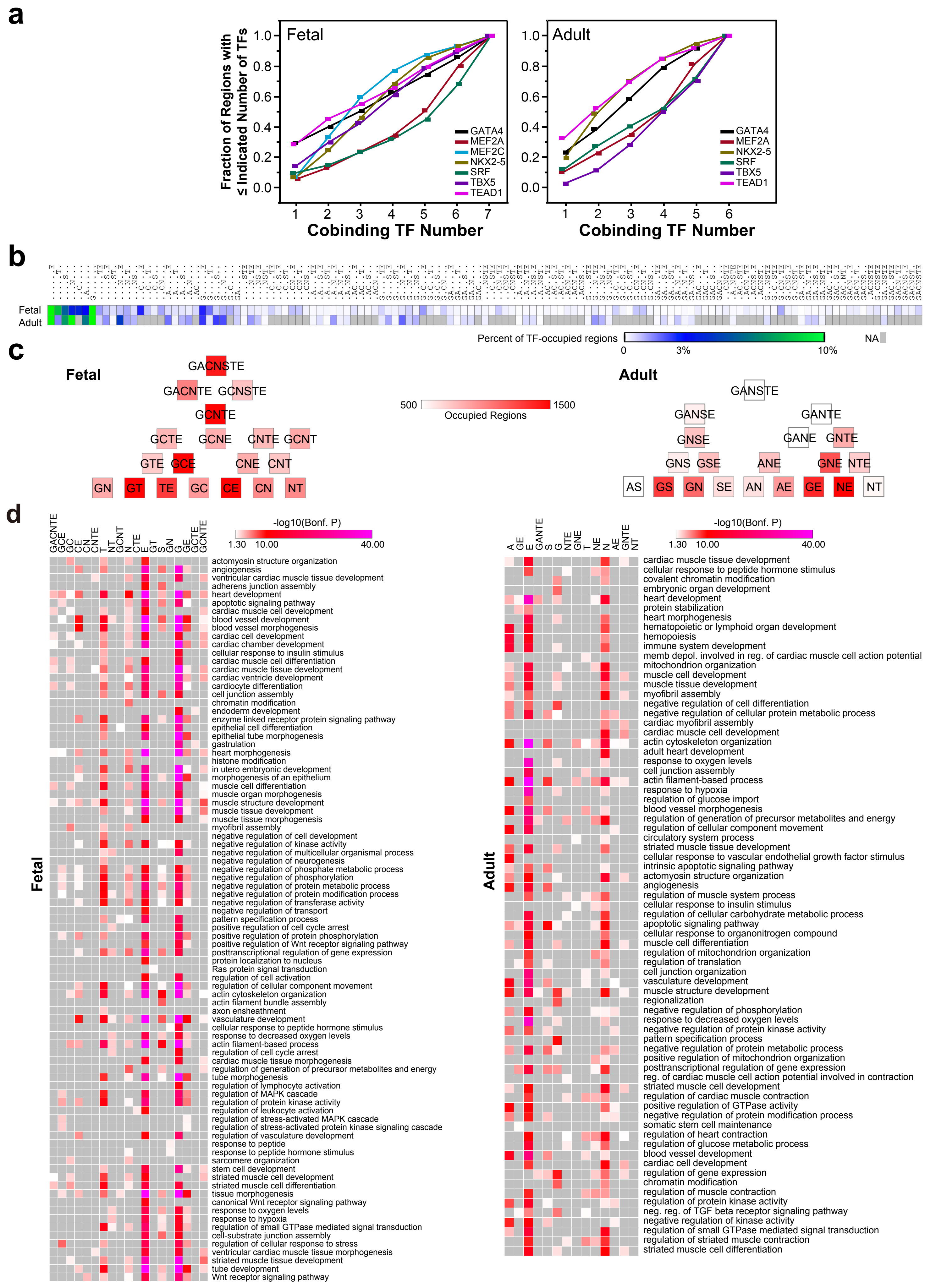

Supplementary Fig. 10, con't

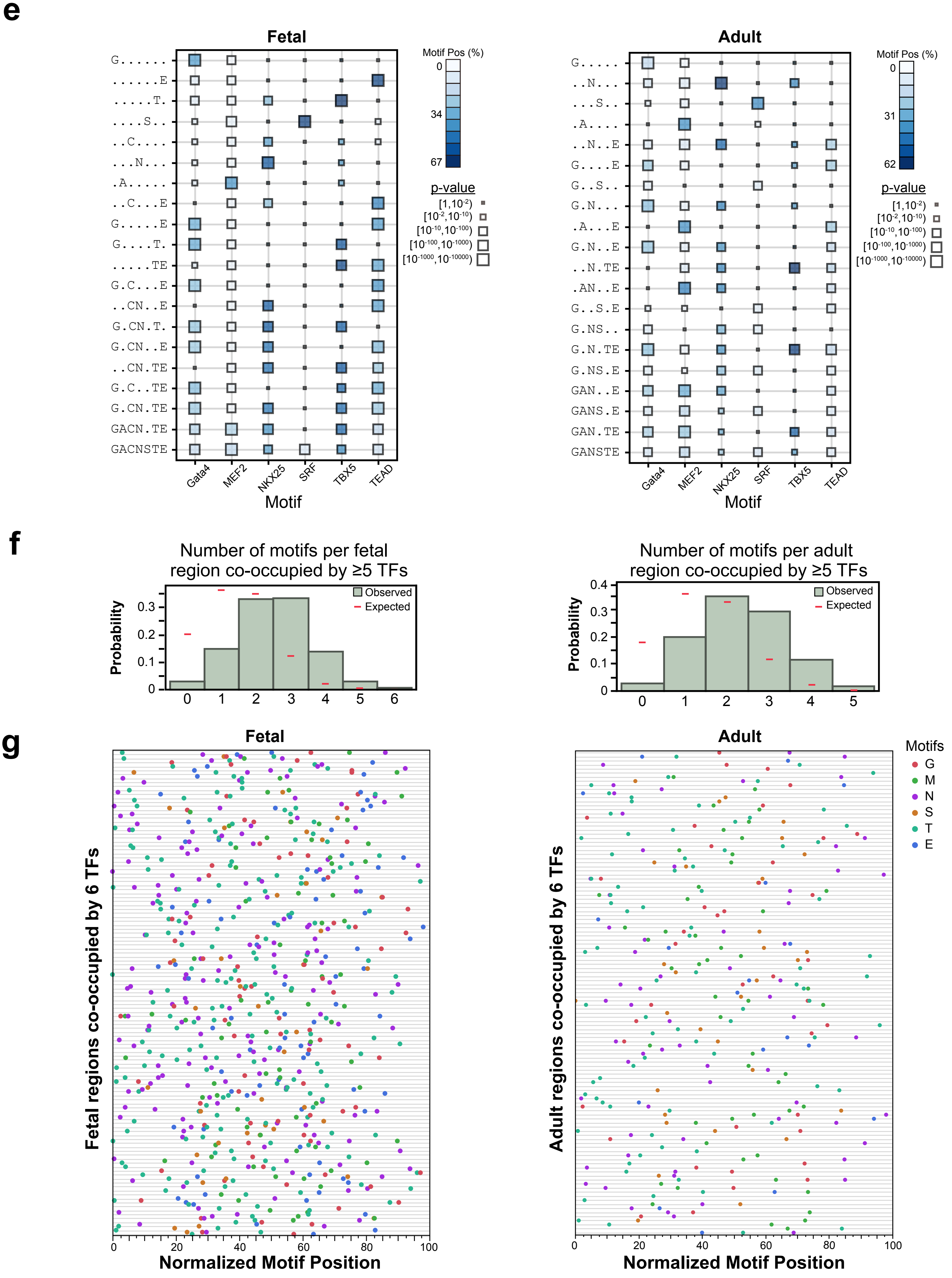

Supplementary Fig. 10, con't

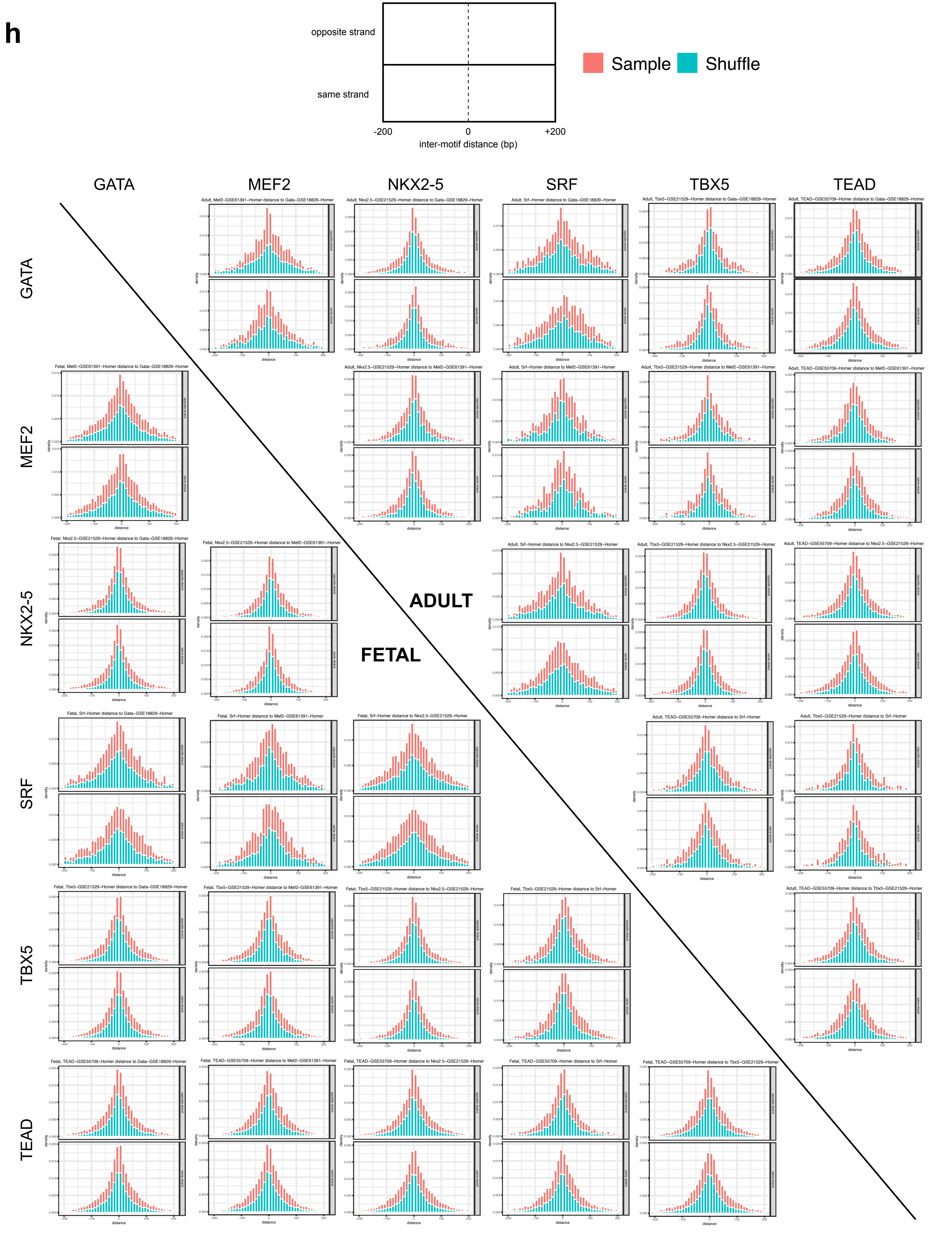

Supplementary Fig. 10, con't

i

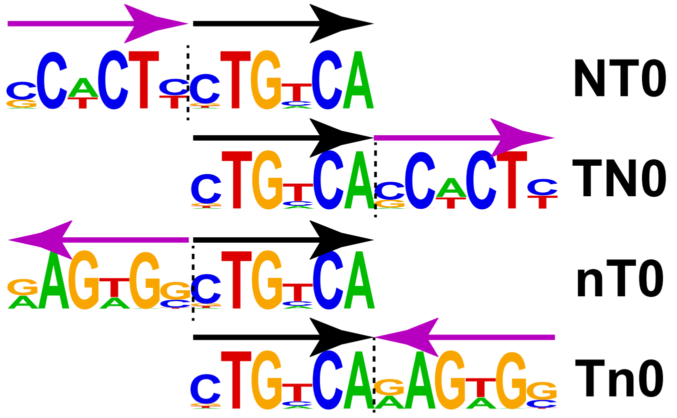

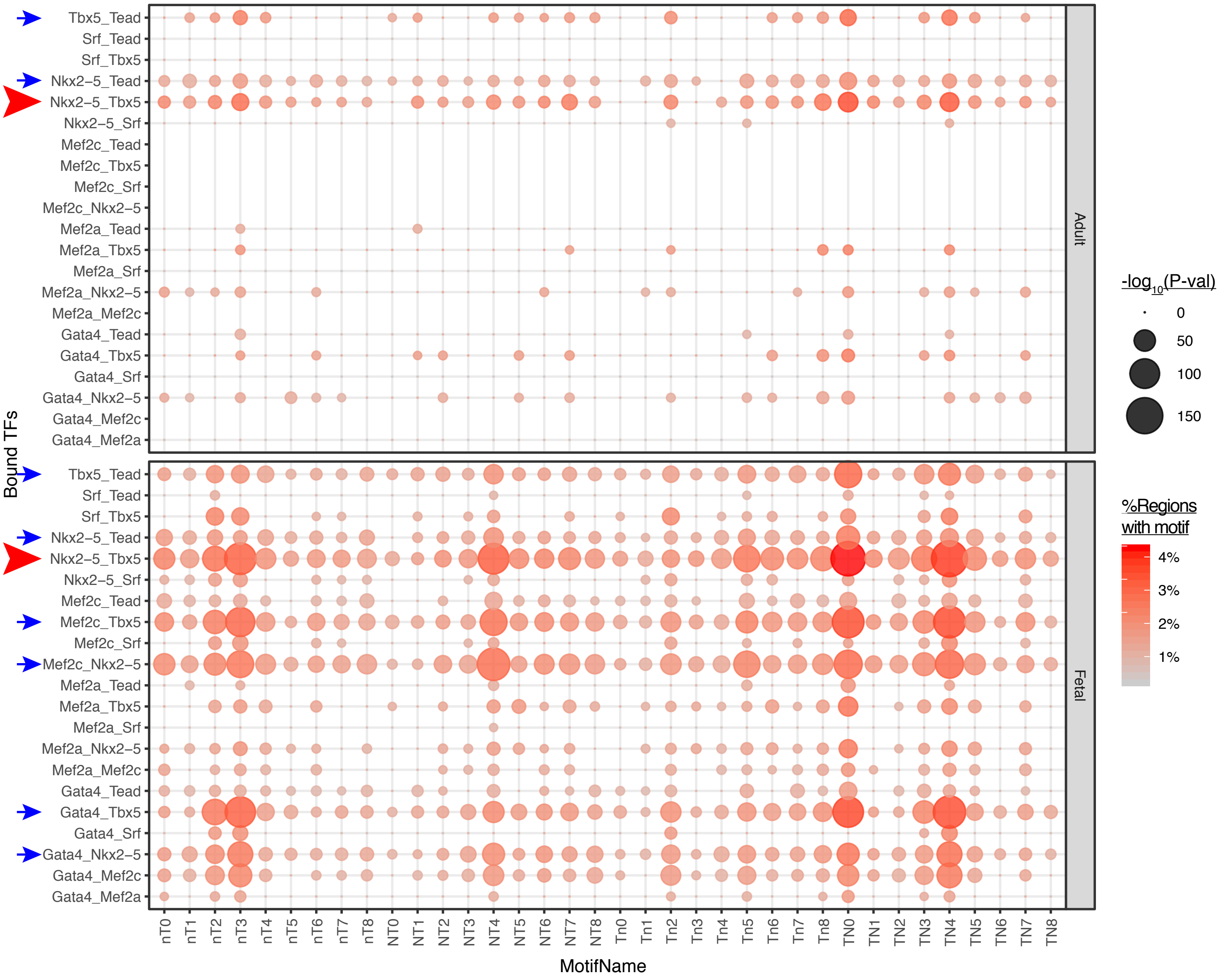

Supplementary Figure 10, con't

j

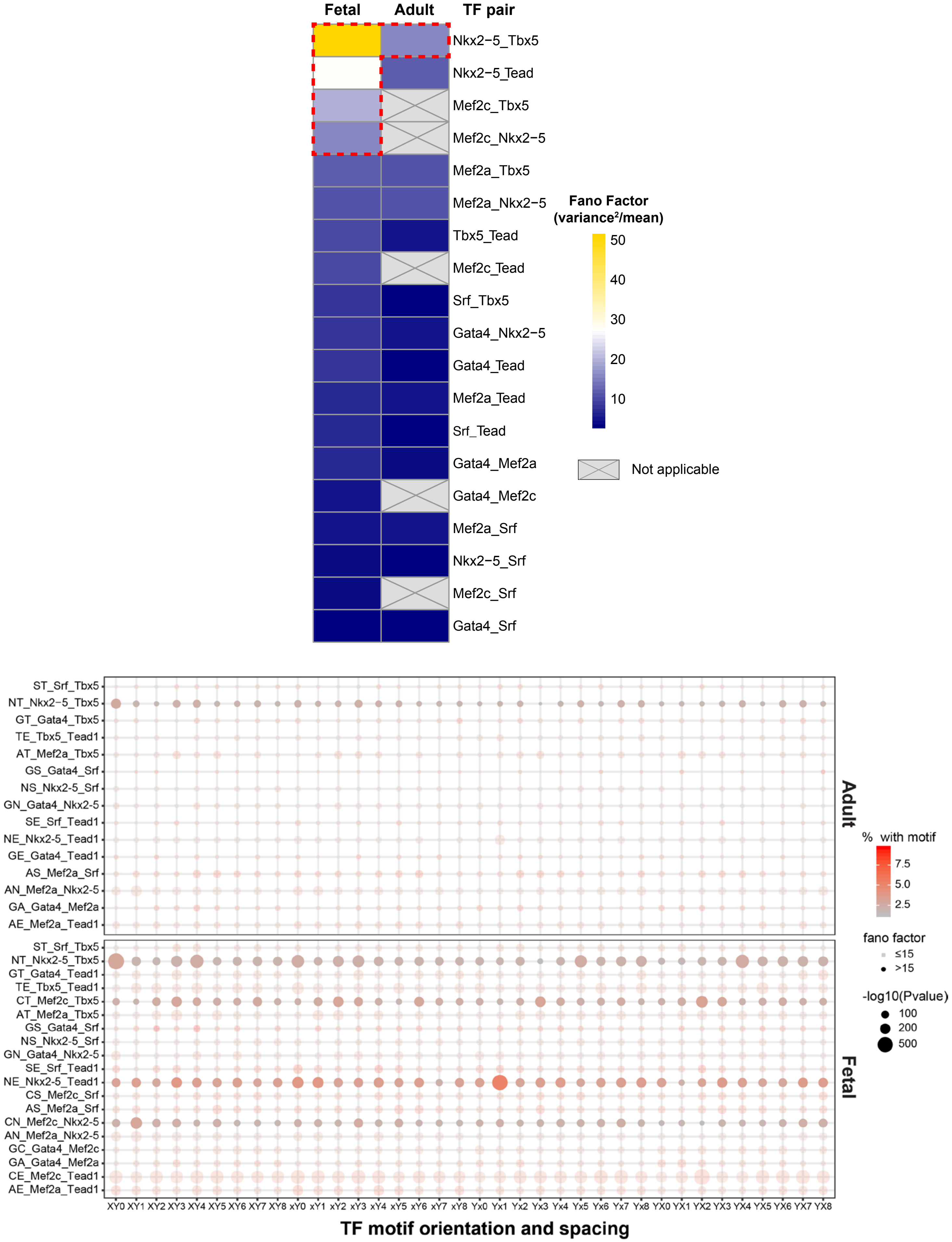

Supplementary Fig. 10, con't

k

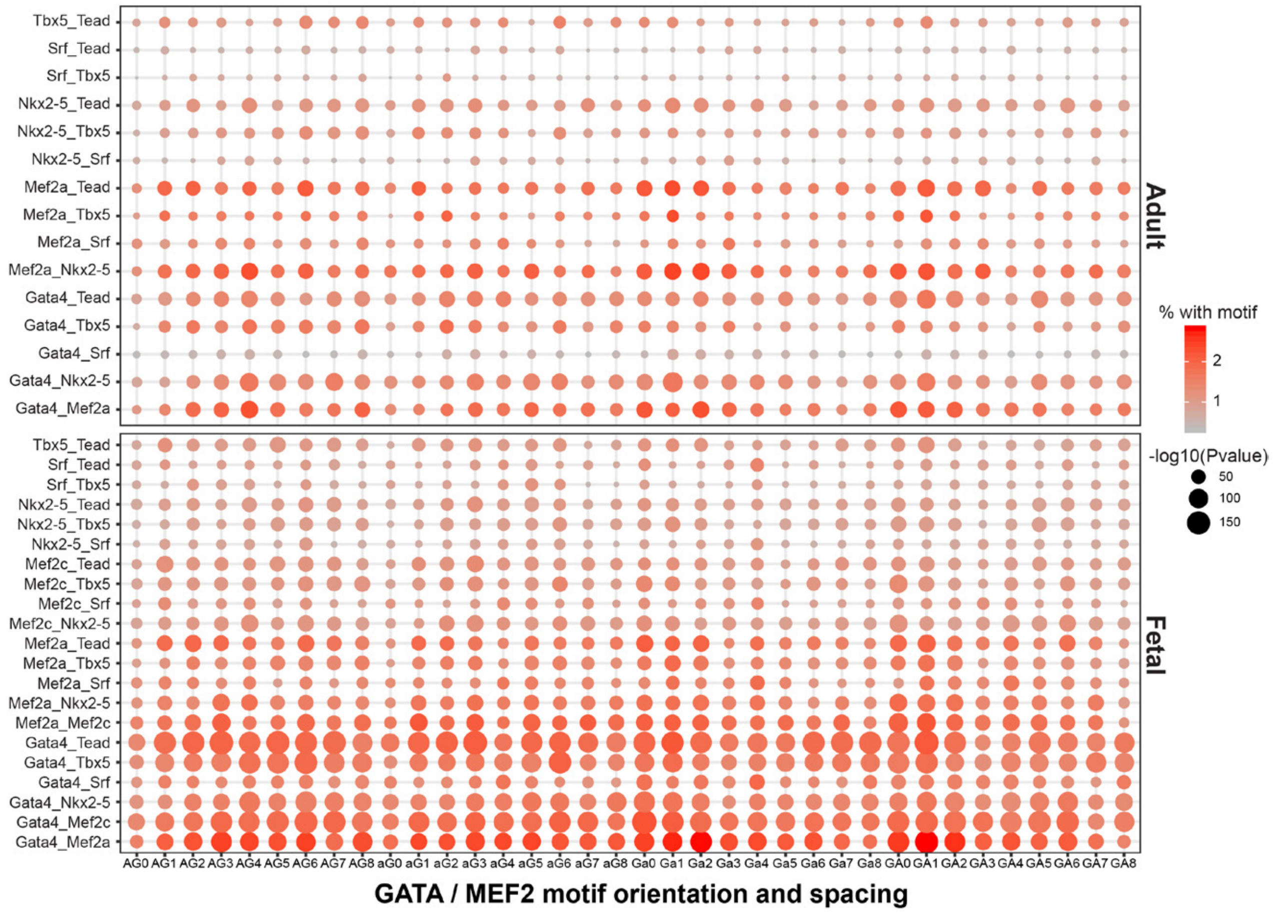

l

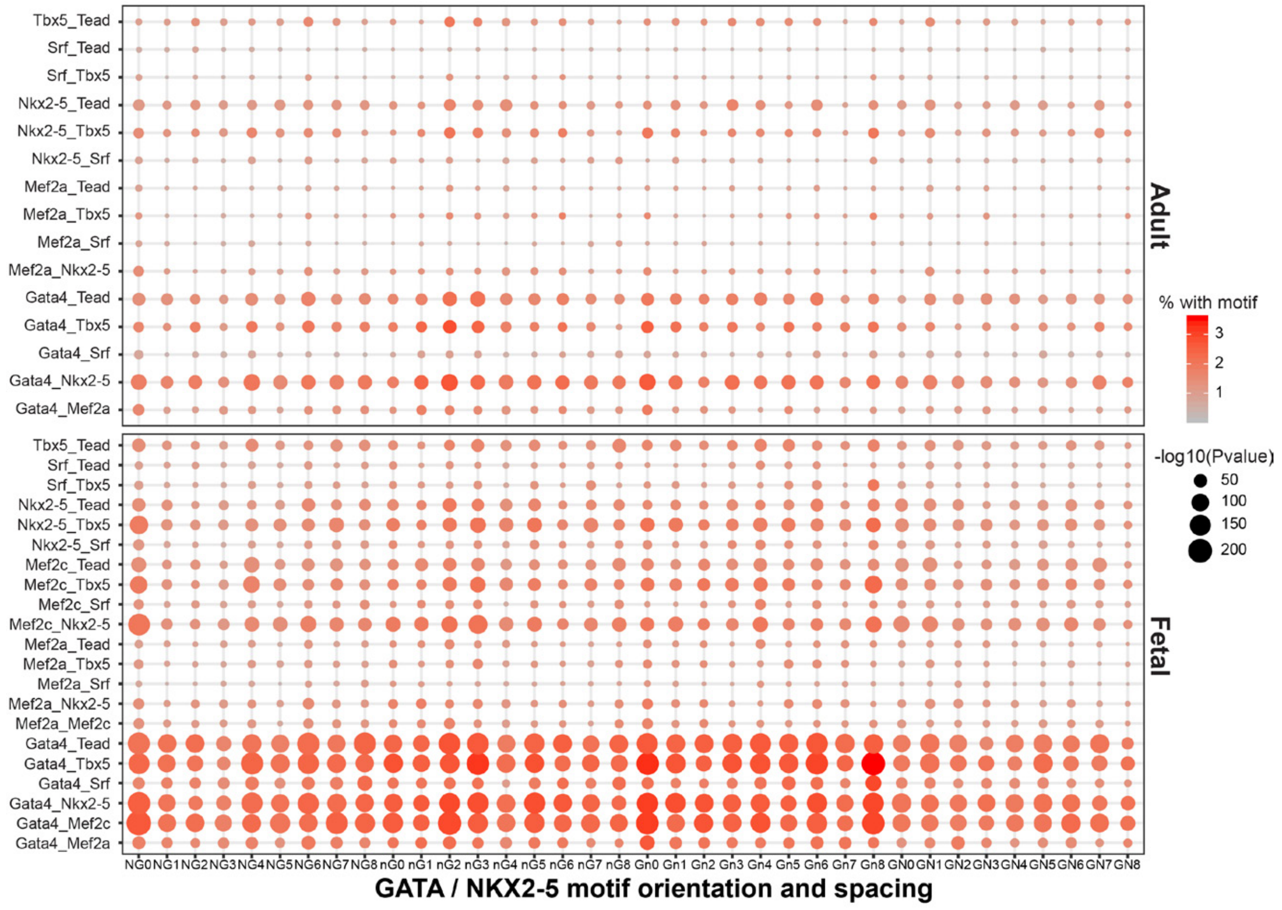

Supplementary Fig. 10, con't

m

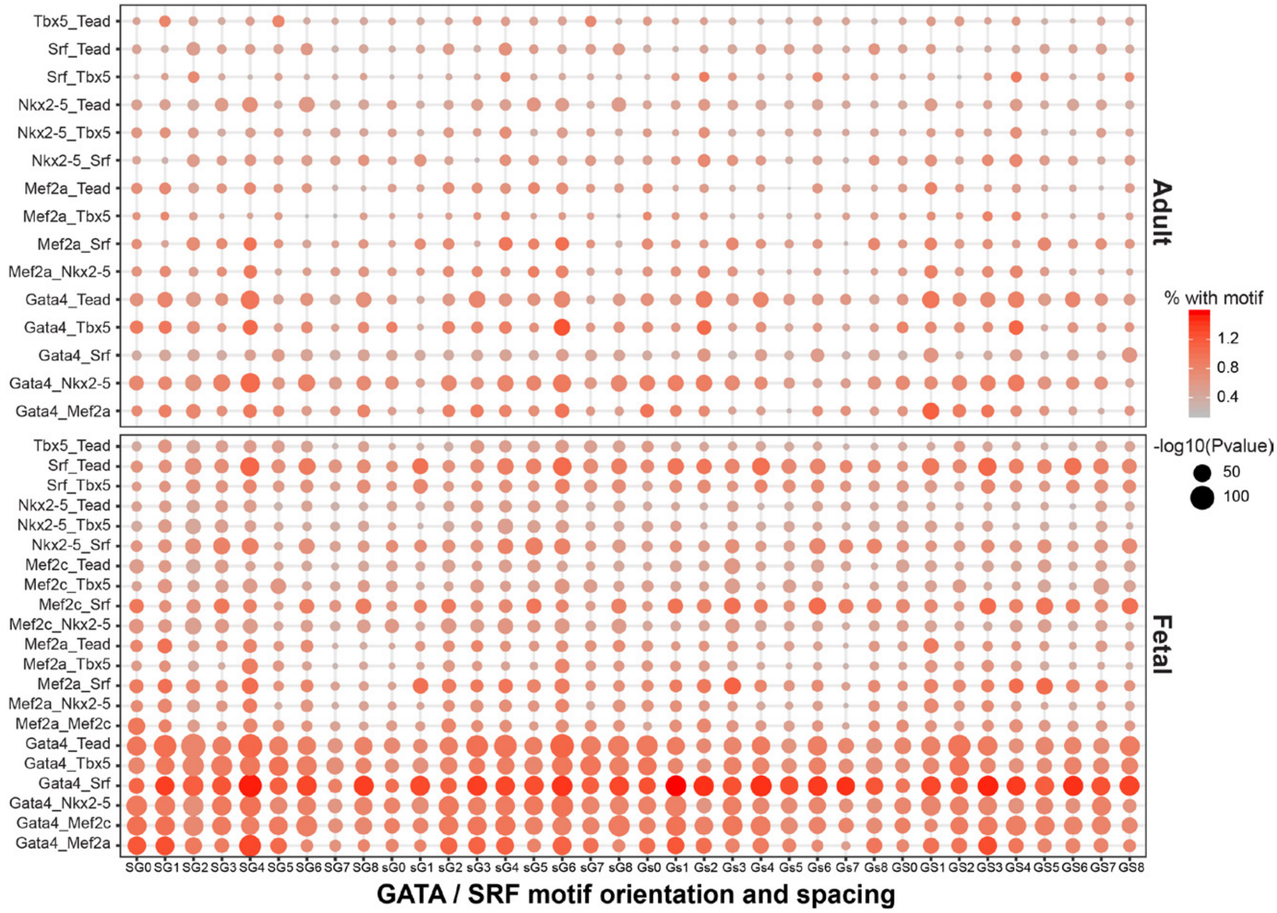

n

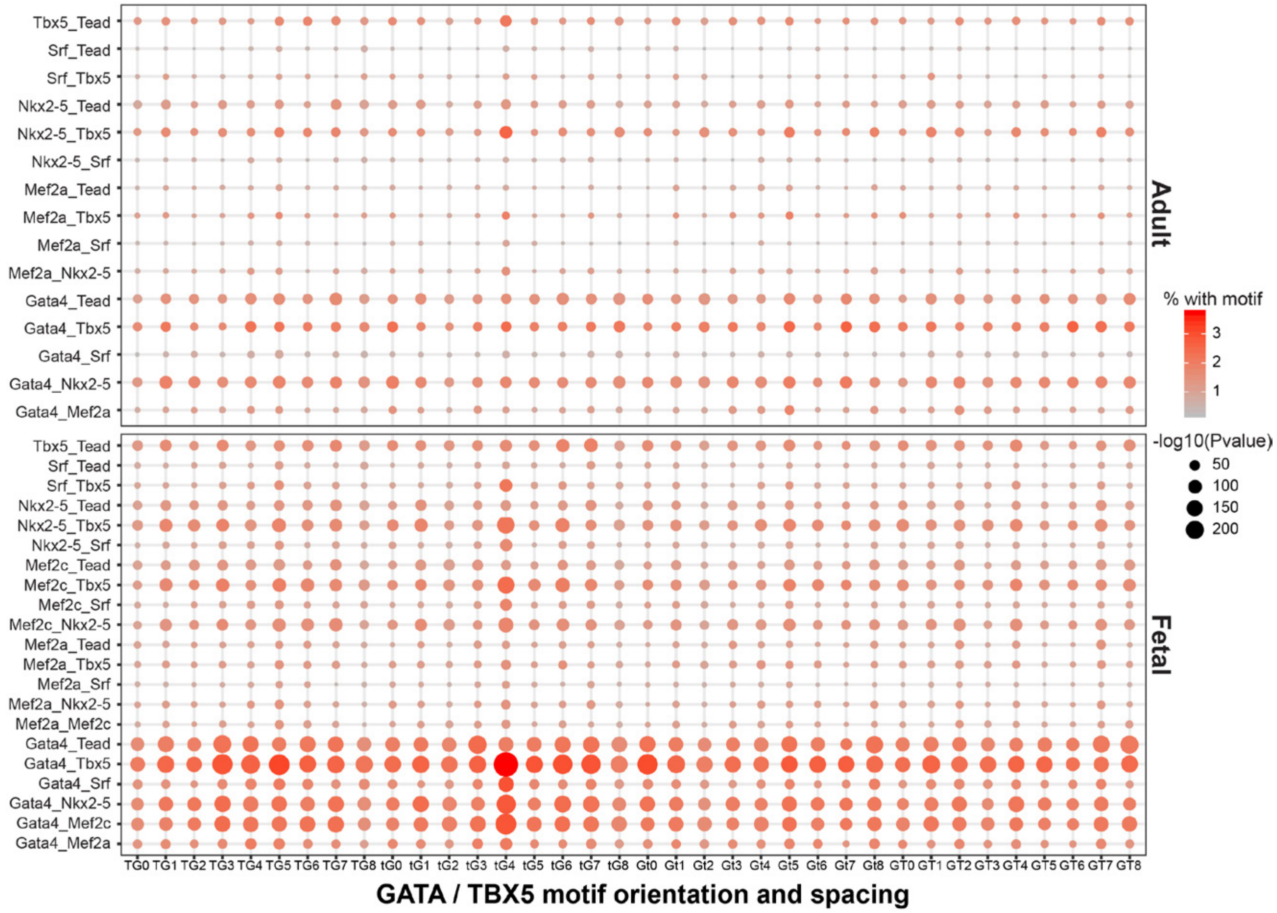

Supplementary Fig. 10, con't

o

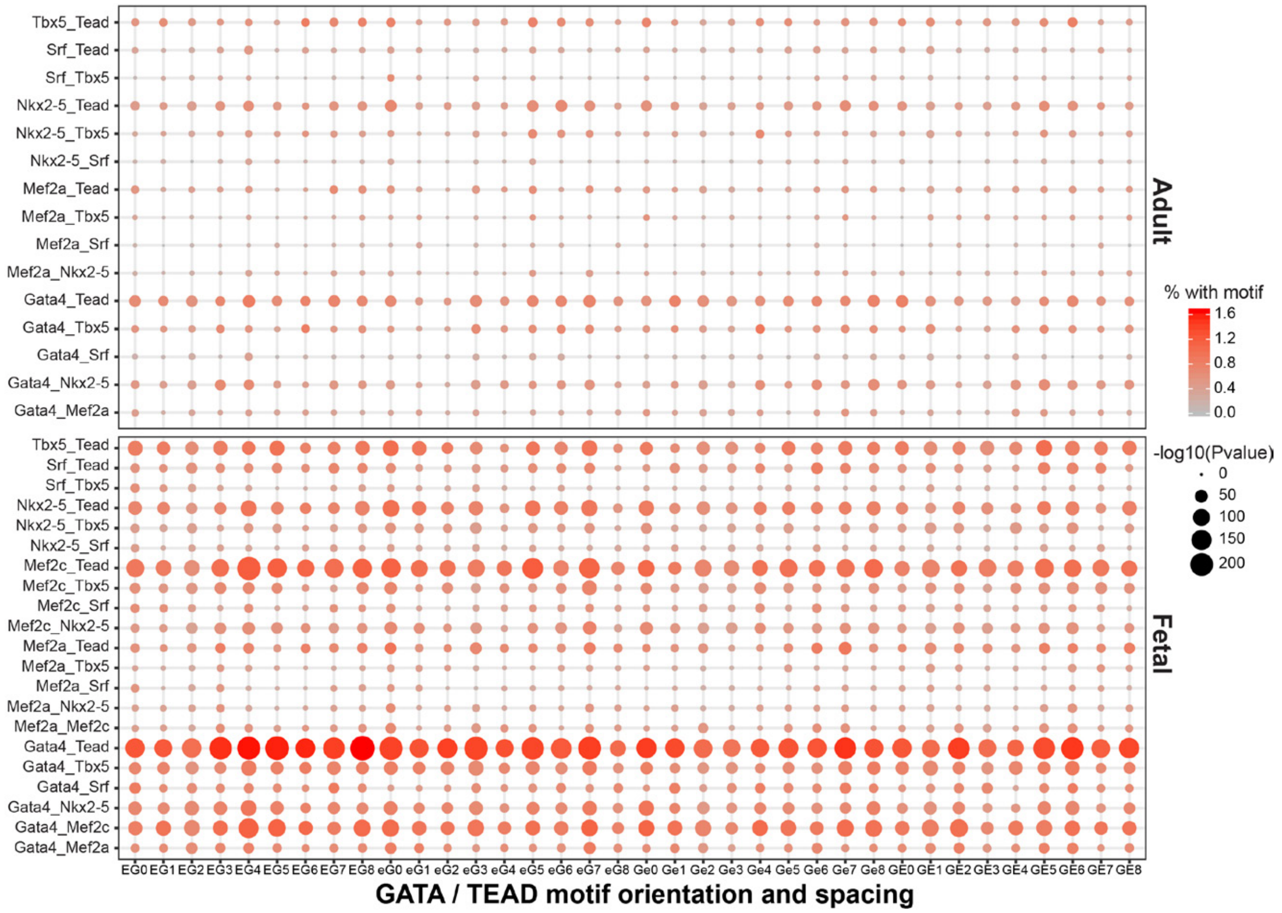

p

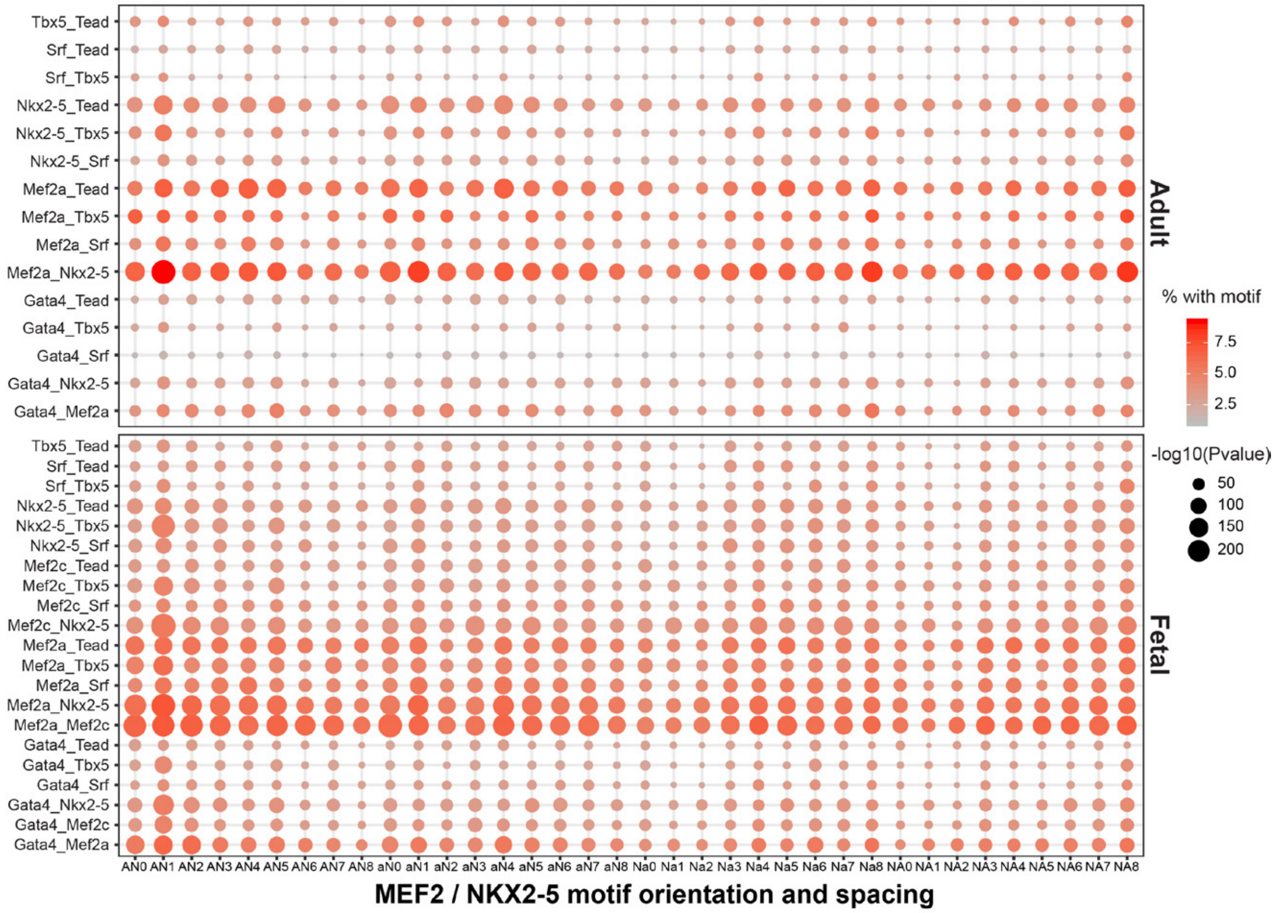

Supplementary Fig. 10, con't

q

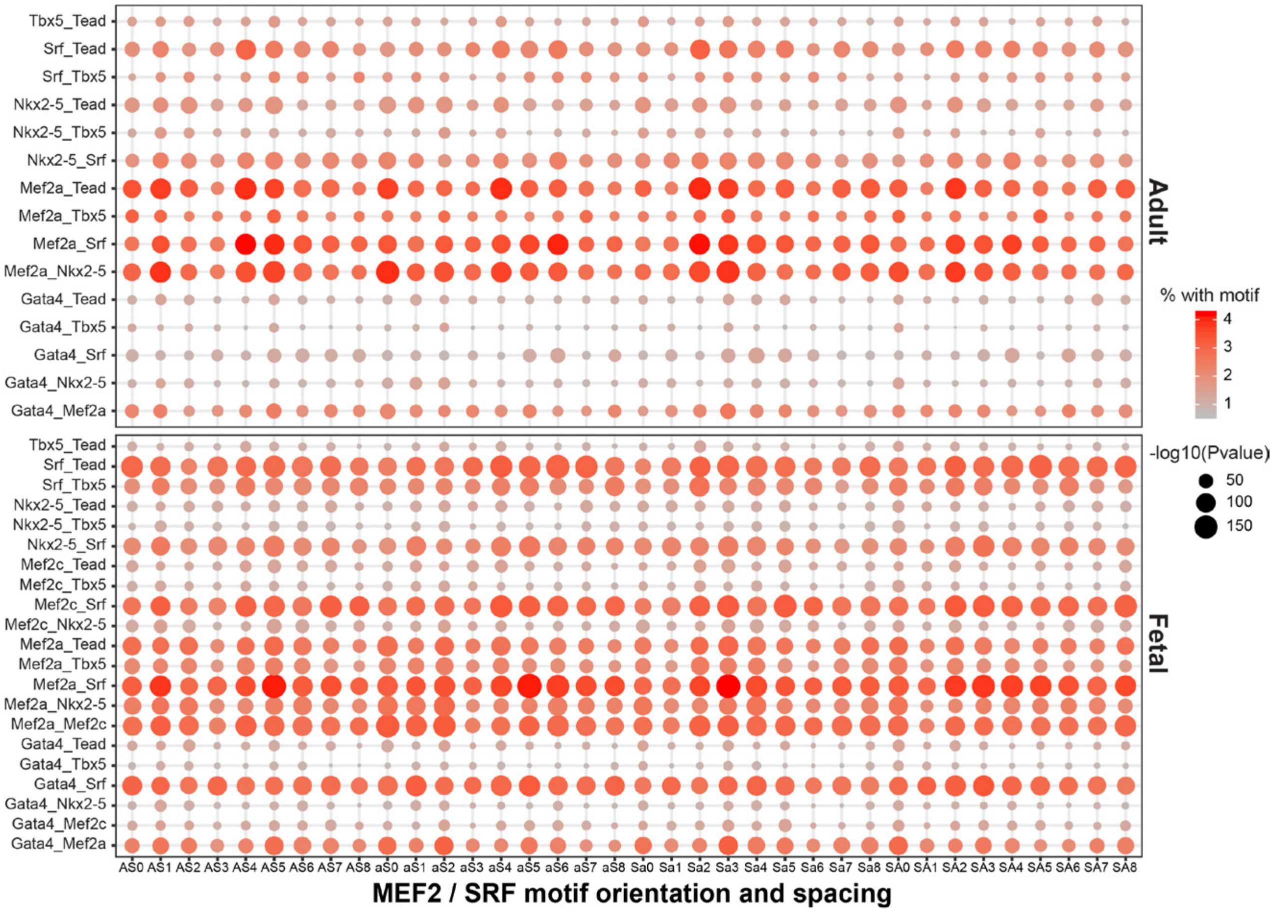

r

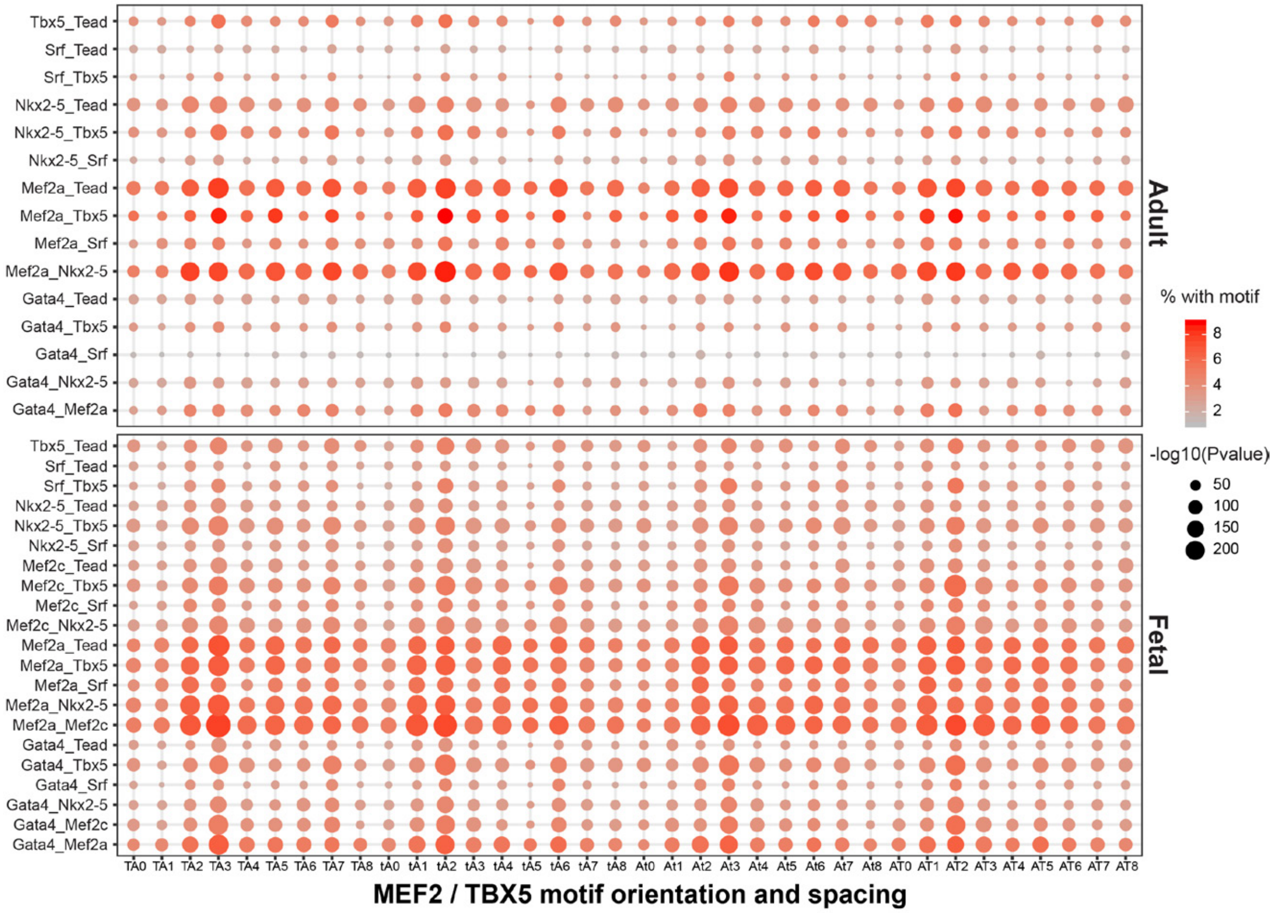

Supplementary Fig. 10, con't

s

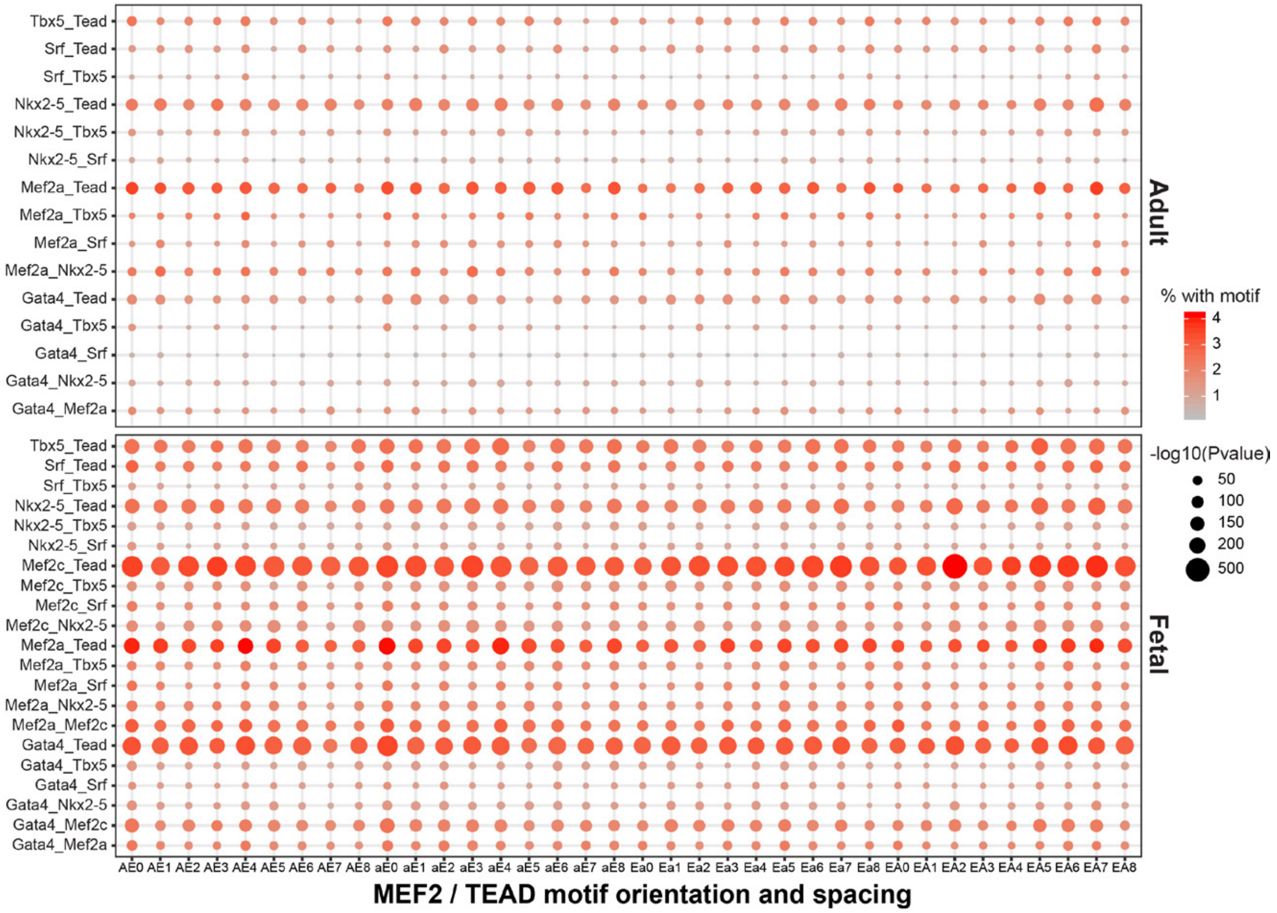

t

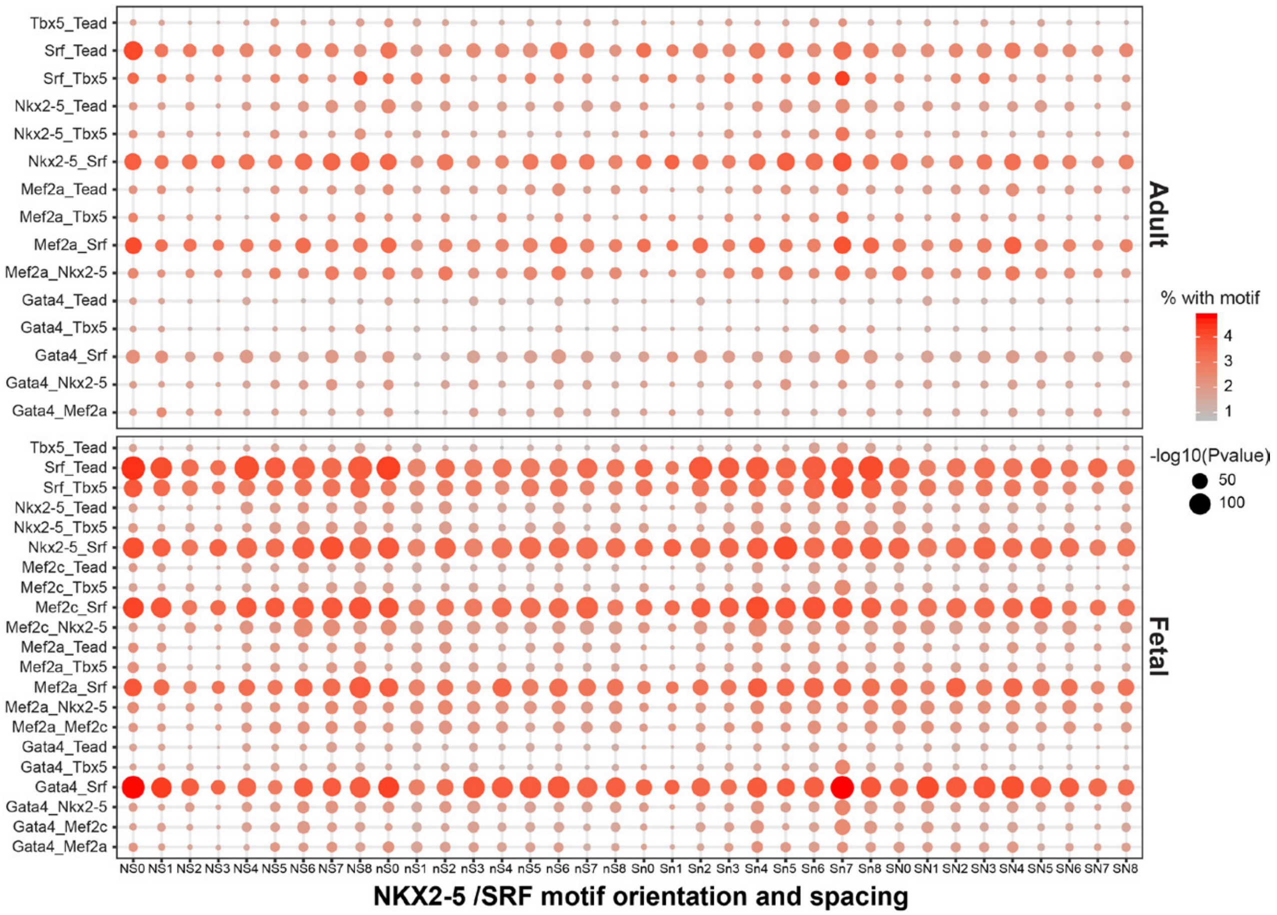

Supplementary Fig. 10, con't

u

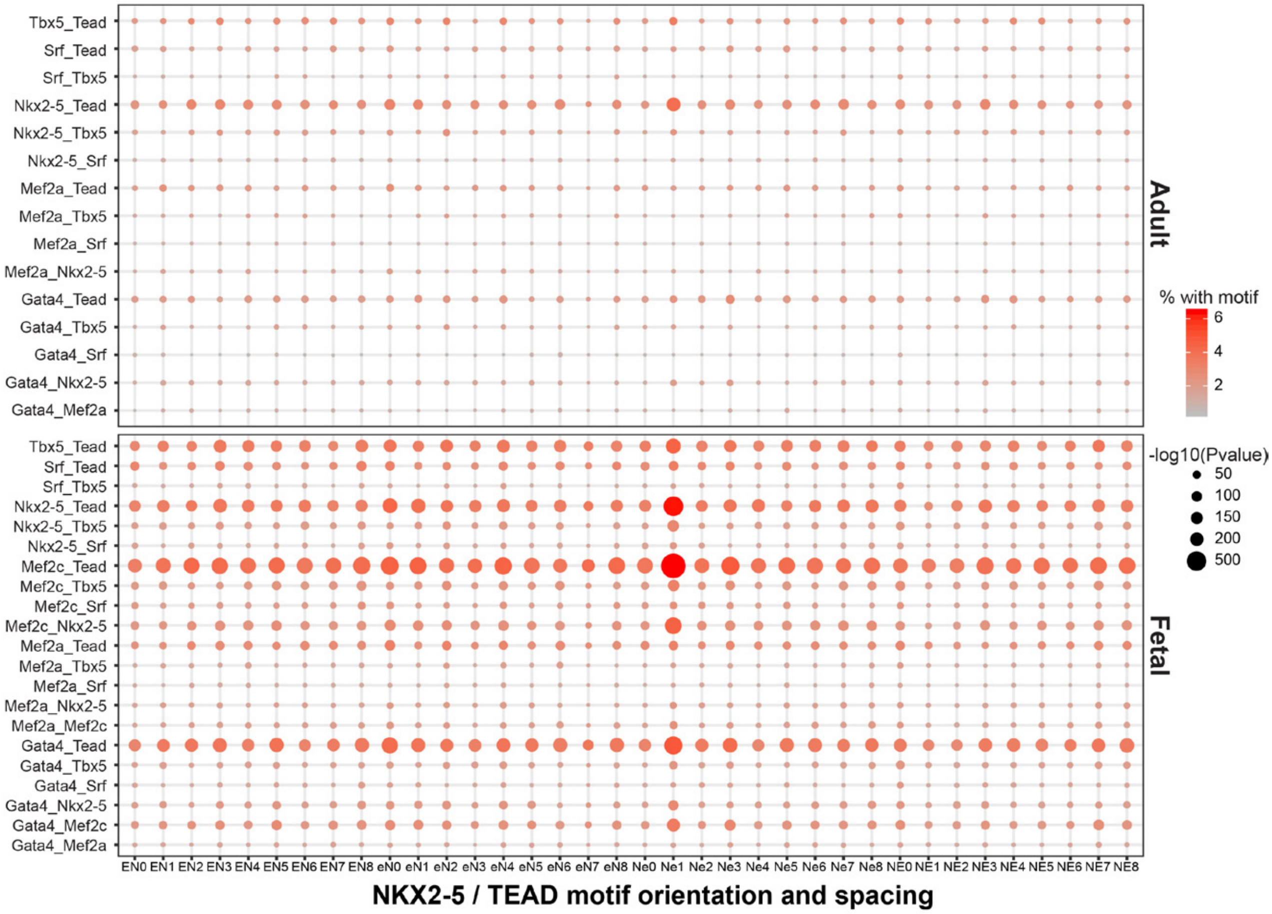

v

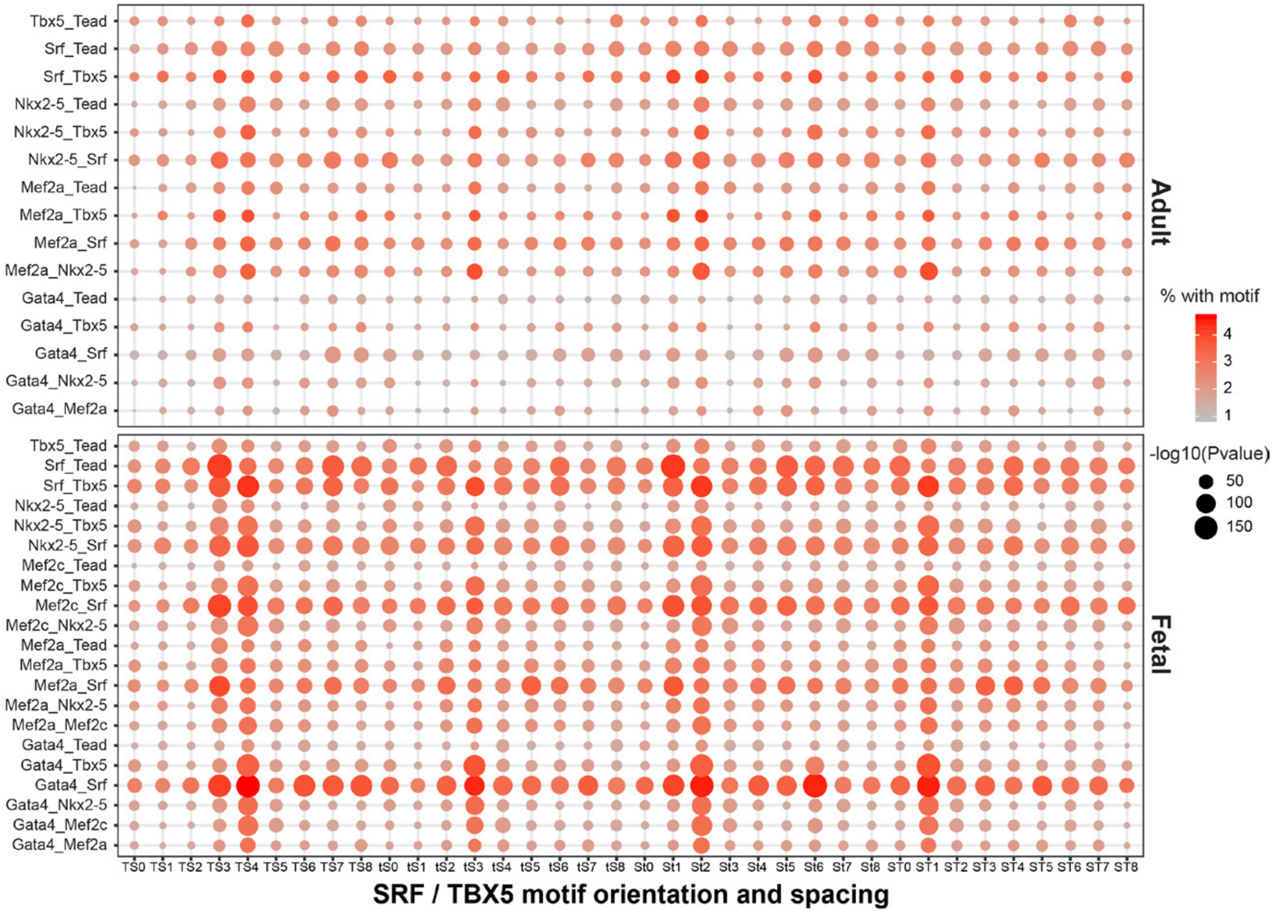

Supplementary Fig. 10, con't

w

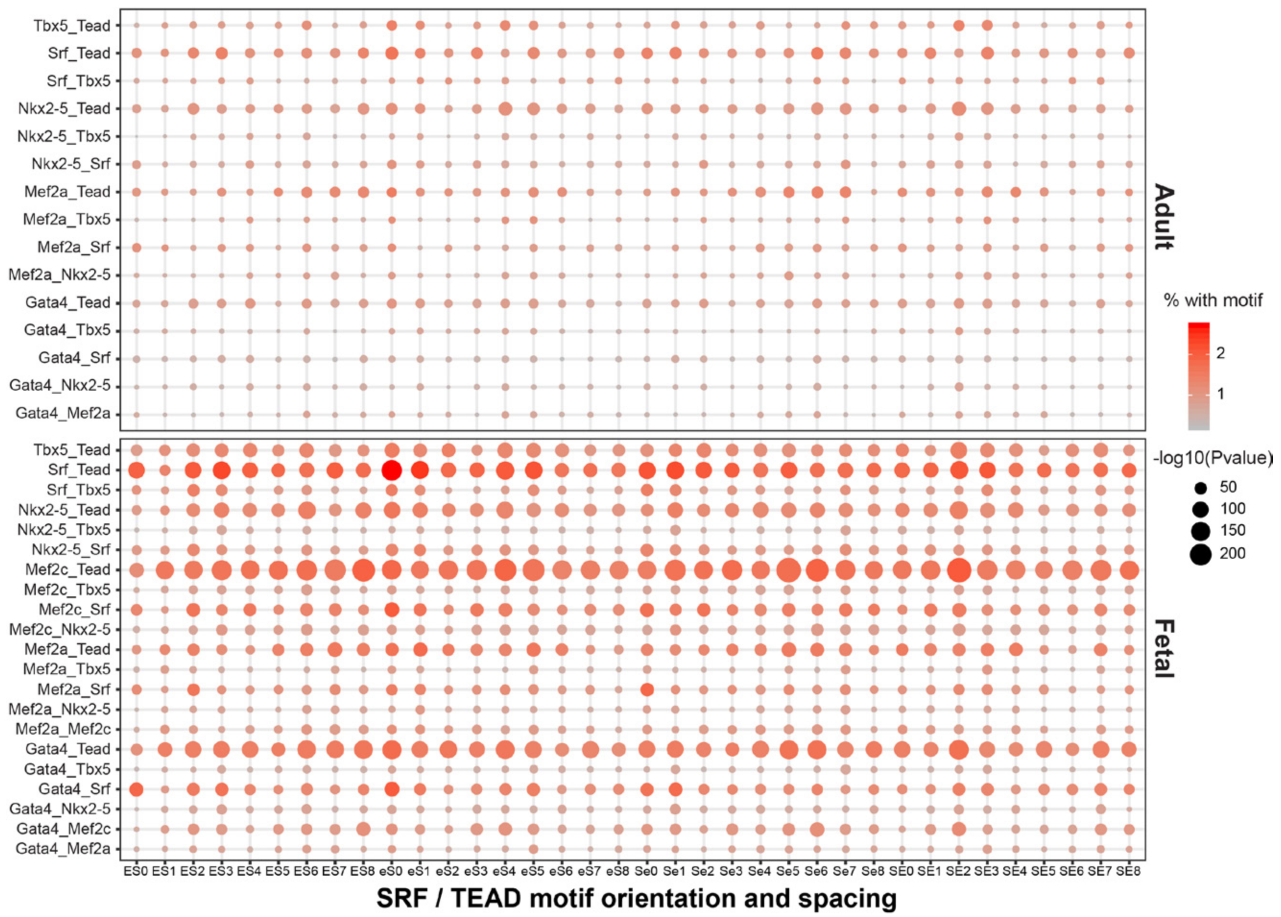

x

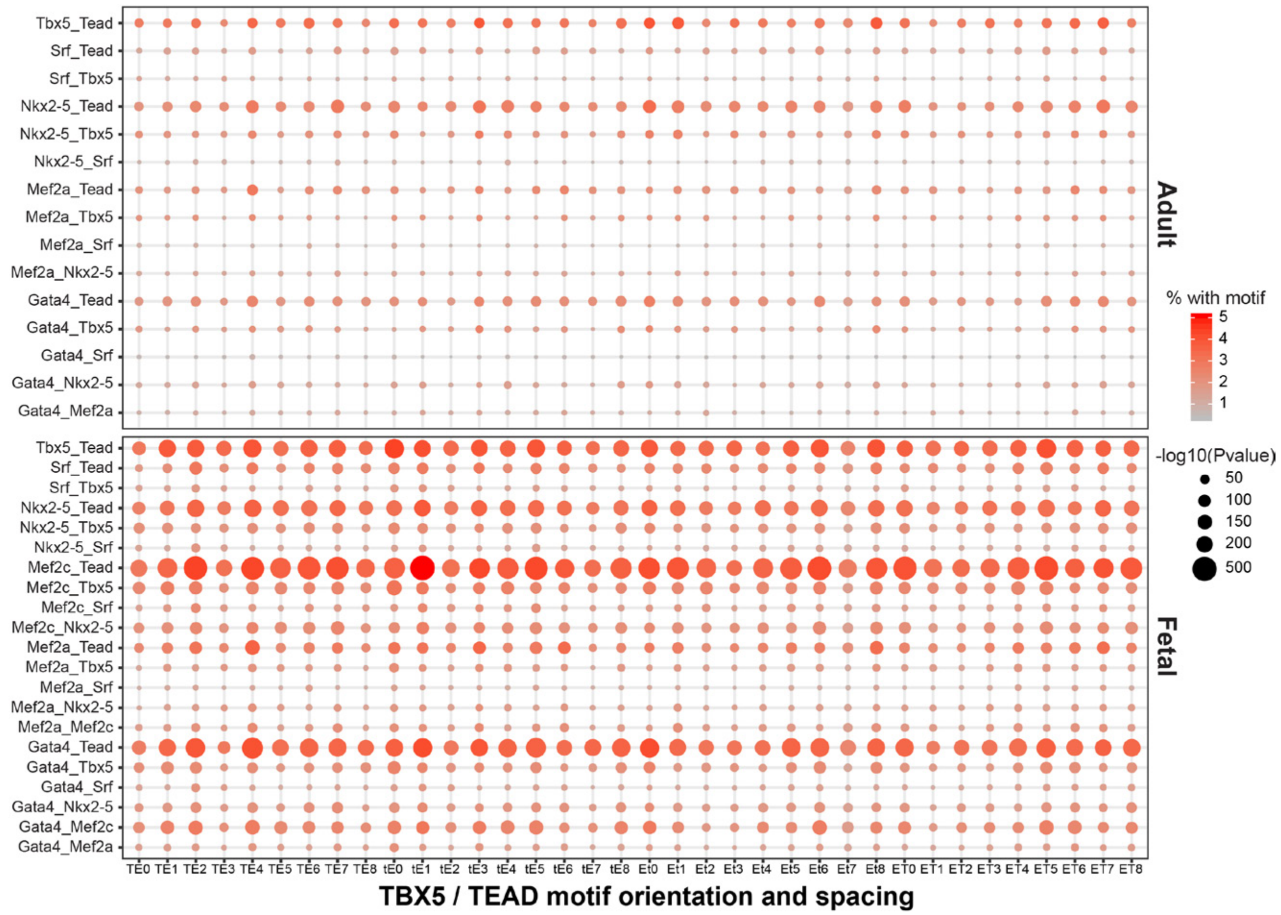

Supplementary Figure 10. Patterns of TF co-occupancy and motif analysis of TF cobound regions.

a. Plot of the fraction of regions with the indicated number of TFs that contain the specified TF. SRF less frequently participated in multiTF co-occupancy in both fetal and adult heart. **b.** Frequency of the 27 fetal (128) and 26 adult (64) possible TF co-occupancy patterns ("cobinding modules") as a percentage of all TF-occupied regions at the same stage. Factors are listed in the order GACNSTE, and '.' indicates absence of the factor. Adult patterns containing 'C' are shown in grey. **c.** Most frequent TF cobinding modules involving two or more different TFs. Color indicates the number of regions for each of the displayed modules. **d.** Biological process gene ontology terms enriched for the top fetal and adult TF modules. The heatmap shows the enrichment p-value for the union of the 5 terms most significant terms identified for each pattern. Grey, P>10-3. **e.** Motif analysis of the most common 20 TF occupancy patterns. Plot shows the p-value and % motif positive regions for GATA, MEF2, NKX2-5, SRF, TBX5, and TEAD motifs within each combination. Each ChIP'd factor enriched for its own motif. ChIP of Fetal MEF2C yielded the NKX2-5 motif. **f.** Distribution of number of TF motifs per region co-occupied by five or more TFs in fetal or adult heart. Red lines indicate median distribution for number of TF motifs in 1000 random permutations of the fetal or adult regions occupied by five or more TFs. **g.** Location of TF motif motifs within fetal or adult regions co-occupied by 6 TFs. A randomly selected subset of these regions are displayed. Each motif is represented by a colored dot. The position of the dot is the normalized distance of the motif from the region's 5' end. **h.** All regions occupied by the indicated pairs of TFs were analyzed for the spacing and strand orientation between the TF's cognate motifs.Fetal and adult regions are shown below and above the diagonal line, respectively. Shuffle indicates 5-fold shuffling of the starting sequences, preserving dinucleotide frequency. No predominant motif orientation or spacing was detected. **i.** All regions occupied by indicated pairs of TFs were analyzed for enrichment of sequence motifs composed of the NKX2-5 and TBX5 6 bp motifs described in Luna-Zurita et al. 2016. The definition of each sequence motif is illustrated by the sequence logos below. The order of the letters specifies the motif order; lower case n indicates that the NKX2-5 motif is on the opposite strand from the TBX5 motif; the number from 0 to 8 indicates the number of random basepairs between the motifs. Regions co-occupied by NKX2-5 and TBX5 (red arrowheads) were most enriched for Nkx2-5 and Tbx5 motifs, especially TN0, TN4 and NT4, as well as nT2 and nT3. Other regions co-occupied by either NKX2-5 or TBX5 and at least one other factor (blue arrows) were also enriched for the same types of Nkx2-5 and Tbx5 motif combinations, likely because of co-occupancy of a subset of these regions by both NKX2-5 and TBX5. **j**. Variation of motif enrichment by motif orientation and spacing. The Fano factor for composite motif enrichment for each TF pair is displayed as a heatmap. The TF pairs with the highest degree of variation based on motif orientation and spacing are outlined by the red dotted line.**k-x.** Comparable motif analysis for all other motif pairs (columns) at all regions co-occupied by the indicated TF pairs (rows). Source data for panels a-f,i-x are provided in SourceData_Supplementary.

Supplementary Fig. 11

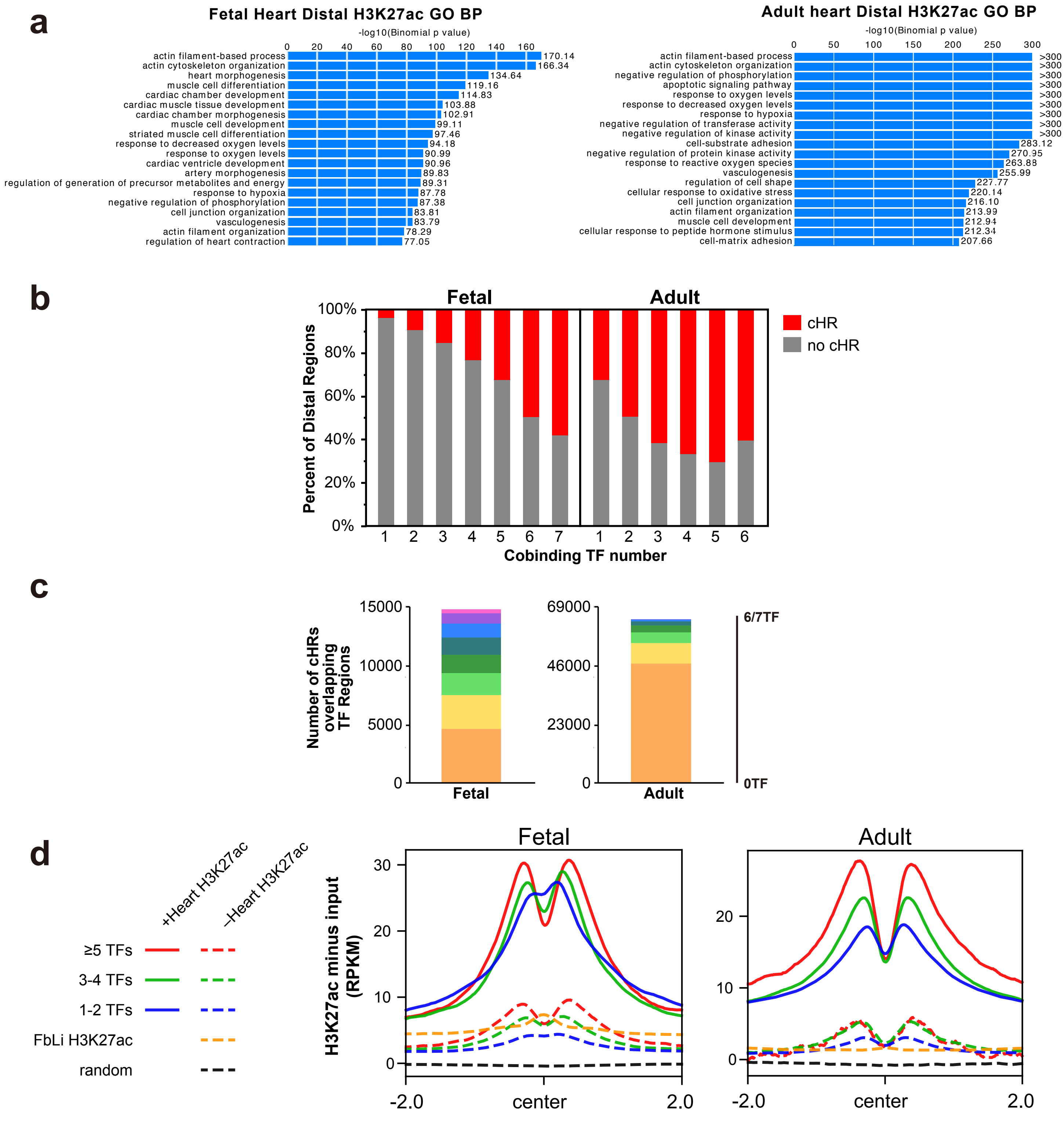

Supplementary Figure 11. Cardiac H3K27ac regions.

a. Gene ontology biological process terms enriched for genes neighboring fetal or adult distal H3K27ac regions. **b.** Overlap of distal TF cobound regions with fixed size with cHR regions. Distal TF cobound regions were set to 500 bp in length (original region center +/- 250 bp). The plot shows the overlap of these 500 bp regions with cHRs. **c.** cHR overlap with TF co-occupied regions. Colors indicate different number of co-occupying TFs, with dark orange indicating no TF co-occupancy (cHR only regions). **d.** H3K27ac signal at TF bound regions, subsetted by whether or not the TF bound region overlaps an region with sufficient H3K27ac signal to be called as a statistically significant peak (+H3K27ac) or not (-H3K27ac). For comparison, the plot includes randomly permuted regions (”random”) or regions with H3K27ac in forebrain or liver but not heart (”FbLi H3K27ac”). Source data for panels b,c are provided in SourceData_Supplementary.

Supplementary Fig. 12

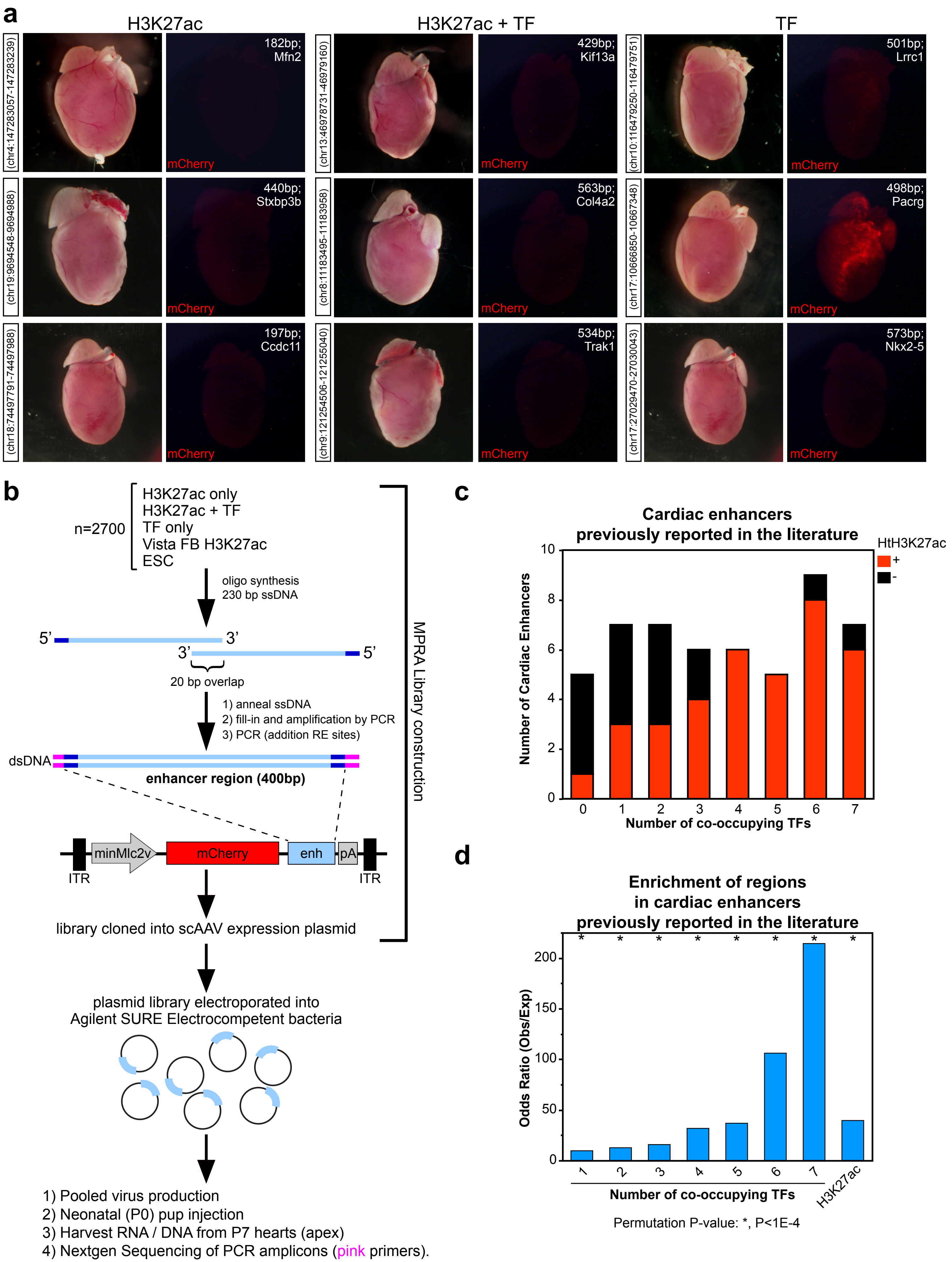

Supplementary Figure 12. AAV-mediated enhancer reporter assays. a. Individual fluorescent reporter assays testing enhancer activity. Darkfield and fluorescent wholemount images for additional regions assayed and classified as either H3K27ac, H3K27ac + TF, or TF. Length (bp) and location of DNA region cloned are stated. **b.** Massively parallel reporter assay (MPRA) workflow. Library of 2700 enhancer regions, comprised of negative control regions (ES and Vista enhancer forebrain H3K27ac regions) and candidate heart enhancers (regions occupied in adult heart by H3K27ac, ≥5 TFs, or H3K27ac and ≥5 TFs), was synthesized as a pool of 5400 single stranded DNA oligonucleotide pairs that were 230 bp long and overlapped by 20 bp. Enhancers were assembled by annealing, primer extension, and PCR amplification. Additional rounds of PCR amplification were carried out to add restriction endonuclese sites (RE) for subsequent plasmid cloning. Library was cloned into a self-complementary AAV (scAAV) for pooled virus production. Reporter vector contains a minimal Mlc2v promoter, an mCherry reporter gene, and an enhancer cloning site located within the reporter gene's 3' untranslated region. Neonatal mouse pups were subcutaneously injected with the AAV-MPRA library and harvested at P7 to test for enhancer activity. Enhancer activity was measured by next generation sequencing to determine enhancer abundance in RNA (transcriptional activity) normalized to DNA. Source data for panels c,d are provided in SourceData_Supplementary.

Supplementary Fig. 13

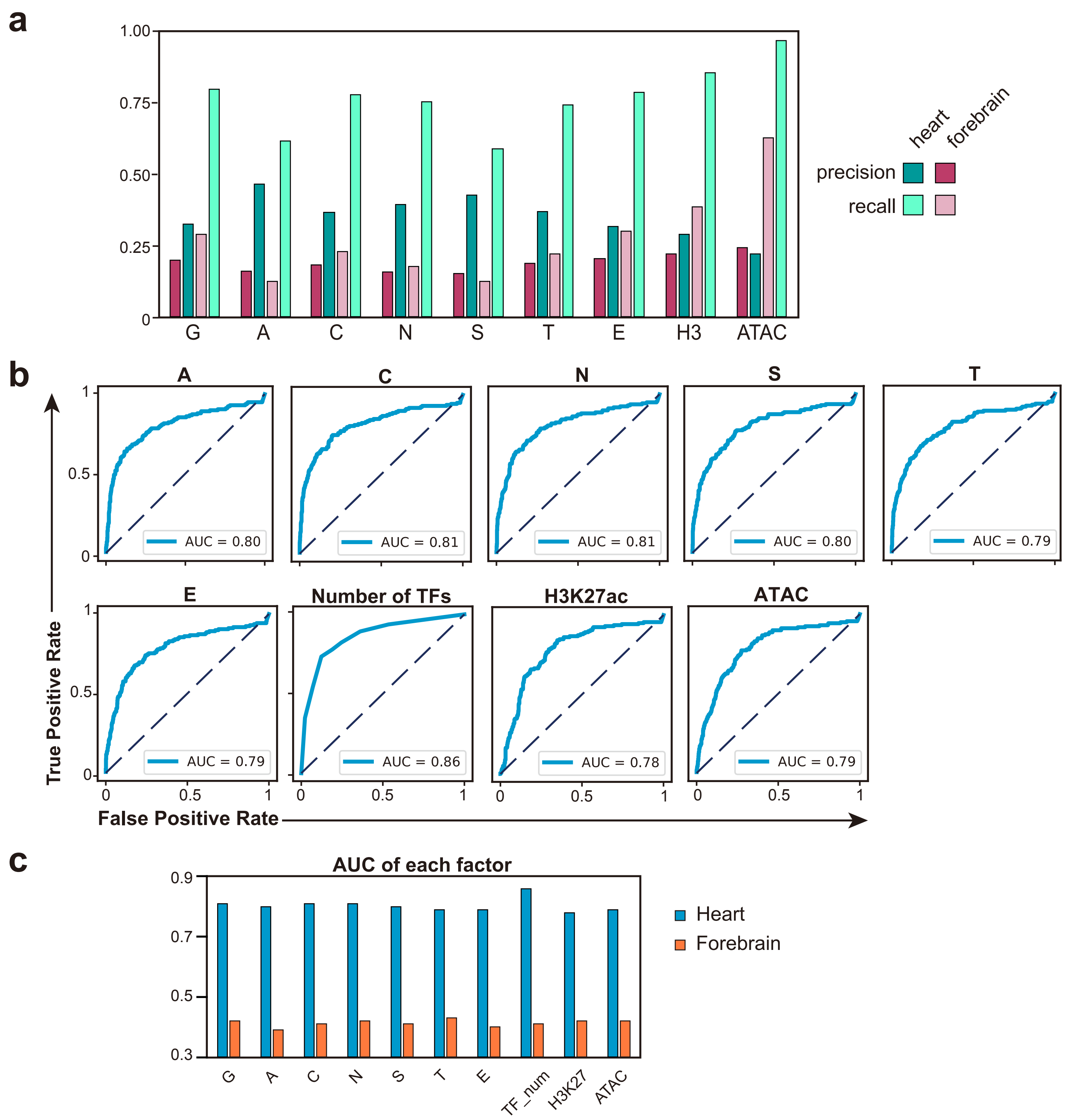

Supplementary Figure 13. Prediction of active cardiac enhancers. a. Precision and recall of individual heart chromatin features for enhancers active in heart or forebrain. **b.** ROC curves show the performance of individual chromatin features to predict active heart enhancers in the VISTA enhancer database. **c.** Prediction accuracy (AUC) for individual heart chromatin feactures for enhancer activity in heart or forebrain. Source data for panels a,c are provided in SourceData_Supplementary.
