# Supplementary material for: A reference map of murine cardiac transcription factor chromatin occupancy identifies dynamic and conserved enhancers
Source: Nat Commun. 2019 Oct 28;10:4907. doi: 10.1038/s41467-019-12812-3 (PMC6817842; doi:10.1038/s41467-019-12812-3)
Supplement: Supplementary file 3 — Description of Additional Supplementary Files [file 41467_2019_12812_MOESM3_ESM.pdf]

Description of Additional Supplementary Files

File Name: Supplementary Data 1
Description: Tabbed excel workbook containing all the summit200 files.

File Name: Supplementary Data 2
Description: Tabbed excel workbook with GO analyses.

File Name: Supplementary Data 3
Description: Tabbed excel workbook with fetal and adult co-bound regions.

File Name: Supplementary Data 4
Description: Tabbed excel workbook of DEGs from this study.

File Name: Supplementary Data 5
Description: Tabbed excel workbook of TF-bound regions adjacent to DEGs found in Tead1-homozygous loss of function.
