# Supplementary material for: A reference map of murine cardiac transcription factor chromatin occupancy identifies dynamic and conserved enhancers
Source: Nat Commun. 2019 Oct 28;10:4907. doi: 10.1038/s41467-019-12812-3 (PMC6817842; doi:10.1038/s41467-019-12812-3)
Supplement: Supplementary file 9 — Reporting Summary [file 41467_2019_12812_MOESM9_ESM.pdf]

nature research

Corresponding author(s): William T. Pu

Last updated by author(s): Jul 26, 2019

Reporting Summary

Nature Research wishes to improve the reproducibility of the work that we publish. This form provides structure for consistency and transparency in reporting. For further information on Nature Research policies, see Authors & Referees and the Editorial Policy Checklist.

Statistics

For all statistical analyses, confirm that the following items are present in the figure legend, table legend, main text, or Methods section.

n/a	Confirmed	
☒	☐	The exact sample size (*n*) for each experimental group/condition, given as a discrete number and unit of measurement
☒	☐	A statement on whether measurements were taken from distinct samples or whether the same sample was measured repeatedly
☐	☒	The statistical test(s) used AND whether they are one- or two-sided<br>*Only common tests should be described solely by name; describe more complex techniques in the Methods section.*
☐	☒	A description of all covariates tested
☐	☒	A description of any assumptions or corrections, such as tests of normality and adjustment for multiple comparisons
☐	☒	A full description of the statistical parameters including central tendency (e.g. means) or other basic estimates (e.g. regression coefficient) AND variation (e.g. standard deviation) or associated estimates of uncertainty (e.g. confidence intervals)
☐	☒	For null hypothesis testing, the test statistic (e.g. *F*, *t*, *r*) with confidence intervals, effect sizes, degrees of freedom and *P* value noted<br>*Give P values as exact values whenever suitable.*
☐	☒	For Bayesian analysis, information on the choice of priors and Markov chain Monte Carlo settings
☐	☒	For hierarchical and complex designs, identification of the appropriate level for tests and full reporting of outcomes
☐	☒	Estimates of effect sizes (e.g. Cohen's *d*, Pearson's *r*), indicating how they were calculated

Our web collection on statistics for biologists contains articles on many of the points above.

Software and code

Policy information about availability of computer code

Data collection: All data analysis was performed using widely available open-access software packages (current versions) described below.

Data analysis

-BioChIP-seq libraries were aligned against the mm9 genome using Bowtie2. Duplicate reads and reads mapping to blacklist regions were removed using Samtools. Peaks were called using MACS2. Reproducible peaks were identified using IDR at IDR_THRESHOLD=0.05 between each set of replicate data files. Deeptools was used to analyze the correlation between BioChIP-seq samples and to generate aggregation plots.
-Motif analysis was performed using Homer. Non-redundant significant motifs were identified by motif clustering using STAMP. Central enrichment of selected motifs within 1000 bp regions centered on peak summits was evaluated with Centrimo.
-To define regions "co-bound" by TFs, the single TF bioChIP-seq peak files were merged using the "mergePeaks" function of Homer.
-Conservation of regions was analyzed using precalculated phastCons 30-way vertebrate scores.
-Gene ontology analysis was performed using GREAT.
-Permutation analysis was performed using regioneR. Regions were randomized 3000-10000 times using the 'randomizeRegions' module. Mappable regions of the genome were defined using 75mer alignment scores in mm9. Xgboost package was used to train an ensemble decision tree model.
-Differentially expressed genes (RNA-seq) were identified using Bioconductor package 'EdgeR' with raw gene counts.
-For ATAC-seq, reads were mapped using Bowtie2. Duplicate reads and reads mapping to blacklisted regions were removed using Samtools. Accessible regions were identified using MACS2.
-For composite motif analysis, motif matrices were generated for all four possible motif orientations with zero to 8 intervening random bases. Matrices and Homer58 were used to determine the enrichment of each composite motifs within regions co-occupied by each pairwise combination of TFs. The variance of enrichment across the 32 possible composite motifs was determined by calculating the Fano factor (variance^2/mean).

All current versions of software packages were used and specific parameters are specified in the methods section.

For manuscripts utilizing custom algorithms or software that are central to the research but not yet described in published literature, software must be made available to editors/reviewers. We strongly encourage code deposition in a community repository (e.g. GitHub). See the Nature Research guidelines for submitting code & software for further information.

Data

Policy information about availability of data

All manuscripts must include a data availability statement. This statement should provide the following information, where applicable:

- Accession codes, unique identifiers, or web links for publicly available datasets
- A list of figures that have associated raw data
- A description of any restrictions on data availability

-Sequencing data for this manuscript, summarized in Supplementary Datas 1, 3 and Supplementary Table 1, has been deposited into NCBI GEO database (GSE124008), which can be reviewed using this link: https://urldefense.proofpoint.com/v2/url?u=https-3A__www.ncbi.nlm.nih.gov_geo_query_acc.cgi-3Facc-3DGSE124008&d=DwIBAg&c=qS4goWBT7poplM69zy_3xhKwEW14JZMSdioCoppxeFU&r=CMWV1alzPmYOimiQcoBihLjlmPH2uRaUjet7jVaCBttBhs6fqrkbUTGbYNA4QXXi&m=74CFVRnbOXksOek8m9wwHpMU3kfk0zweIjNFlDtZQLw&s=Q7rzbA_8MH8x2VbEDfIp1RxWUjMCddq4CZMyYMOgeY0&e=
Enter token qvafqoaenvsvfuf into the box
-All bioChIP-seq data can be visualized using this link: https://genome.ucsc.edu/cgi-bin/hgTracks?db=mm9&lastVirtModeType=default&lastVirtModeExtraState=&virtModeType=default&virtMode=0&nonVirtPosition=&position=chr12%3A57803991%2D57810533&hgsid=741360699_YDByfTyaf1JIisQSpA9h5ELj6ZTO

-Data can also be accessed via the Cardiovascular Development Consortium (CvDC) server (https://b2b.hci.utah.edu/gnomex) (sign in as guest).

Field-specific reporting

Please select the one below that is the best fit for your research. If you are not sure, read the appropriate sections before making your selection.

☒ Life sciences ☐ Behavioural & social sciences ☐ Ecological, evolutionary & environmental sciences

For a reference copy of the document with all sections, see nature.com/documents/nr-reporting-summary-flat.pdf

Life sciences study design

All studies must disclose on these points even when the disclosure is negative.

Sample size	BioChIP-seq data was analyzed from biological duplicate samples. Reproducible peaks were identified using IDR (ENCODE standards) at IDR_THRESHOLD=0.05 between each set of replicate data files. Data used for all downstream analyses utilized a single TF bioChIP-seq file generated from IDR and further processed so that each IDR peak was represented by the MACS2 summit ± 100 bp of the individual replicate with the greatest peak intensity. Cardiomyocyte-specific expression data (RNA-seq) generated in this study was performed using an n=3.
Data exclusions	No data was excluded from this study.
Replication	All BioChIP-seq datasets generated in this study were performed in biological replicates. Each experiment was highly reproducible (shown in Figure 1a) with the highest similiarity between the biological duplicates (Spearman coefficient).

Randomization	No samples were randomized. All fetal samples were harvested at embryonic day 12.5 and ventricles were dissected away from other cardiac tissues (atrioventricular cushion, atria, etc.). Each fetal biological replicate sample contained between 95-105 pooled hearts from multiple litters, which were attained through multiple matings with different double homozygous tg males (_fb/fb;BirA/BirA) mated to 'wildtype' Swiss Webster females. All adult samples were harvested at 6 weeks postnatal and each bioChIP-seq sample contained both female and male (littermates) heart ventricle apexes (distal 1/3). Adult heart tissue for bioChIP-seq samples was generated through double homozygous tg males (_fb/fb;BirA/BirA) mated to 'wildtype' Swiss Webster females and the heterozygous progeny were used.
Blinding	Blinding was not relevant to the majority of our study, as all data is non-biased next-generation sequencing. Blinding was only utilized when echos were performed by a researcher for functional analysis of the transgenic mouse lines.

Reporting for specific materials, systems and methods

We require information from authors about some types of materials, experimental systems and methods used in many studies. Here, indicate whether each material, system or method listed is relevant to your study. If you are not sure if a list item applies to your research, read the appropriate section before selecting a response.

Materials & experimental systems

n/a	Involved in the study
☐	☒ Antibodies
☒	☐ Eukaryotic cell lines
☒	☐ Palaeontology
☐	☒ Animals and other organisms
☒	☐ Human research participants
☒	☐ Clinical data

Methods

n/a	Involved in the study
☐	☒ ChIP-seq
☒	☐ Flow cytometry
☒	☐ MRI-based neuroimaging

Antibodies

Antibodies used	Gapdh Invitrogen cat# PA1-16777<br>Gata4 Santa Cruz sc-123X<br>Mef2a Invitrogen cat# PA5-27380<br>Mef2c abcam cat# ab211493<br>Nkx2-5 Santa Cruz sc-8697X<br>Tbx5 Santa Cruz sc-17866X<br>Tead1 BD Biosciences cat# 610923<br>WES Anti-Rabbit HRP detection module #DM001<br>WES Anti-mouse HRP detection module #DM002<br>WES Anti-Goat HRP detection module #DM006
Validation	All antibodies have been validated by manufacturers. Tead1 was further validated by Lin, Z. et al. Acetylation of VGLL4 Regulates Hippo-YAP Signaling and Postnatal Cardiac Growth. Dev. Cell 39, 466–479 (2016).

Animals and other organisms

Policy information about studies involving animals; ARRIVE guidelines recommended for reporting animal research

Laboratory animals	This study includes the use of laboratory mice. All mice used were from a mixed B6/SW strain background and when feasible, (adult bioChIP-seq) both female and male samples were combined. Echos (Supplementary Fig. 2) were performed on both female and male mice.
Wild animals	The study did not involve wild animals.
Field-collected samples	The study did not contain samples collected from the field.
Ethics oversight	Mouse husbandry and procedures were performed under the approval and observation of the Boston Children's Hospital International Animal Care and Use Committee.

Note that full information on the approval of the study protocol must also be provided in the manuscript.

ChIP-seq

Data deposition

☒ Confirm that both raw and final processed data have been deposited in a public database such as GEO.

☒ Confirm that you have deposited or provided access to graph files (e.g. BED files) for the called peaks.

Data access links
May remain private before publication.

https://urldefense.proofpoint.com/v2/url?u=https-3A__www.ncbi.nlm.nih.gov_geo_query_acc.cgi-3Facc-3DGSE124008&d=DwIBAg&c=qS4goWBT7poplM69zy_3xhKwEW14JZMSdioCoppxeFU&r=CMWV1alzPmYOimiQcoBihLjlmPH2uRaUjet7jVaCBttBhs6fqrkbUTGbYNA4QXXi&m=74CFVRnbOXksOek8m9wwHpMU3kfk0zweIjNFlDtZQLw&s=Q7rzbA_8MH8x2VbEDfIp1RxWUjMCddq4CZMyYMOgeY0&e=

Files in database submission

Adult Heart Apex MEF2A 1
Adult Heart Apex MEF2A 2
Adult Heart Apex MEF2A-input
Adult Heart Apex NKX25 1
Adult Heart Apex NKX25 2
Adult Heart Apex NKX25-input
Adult Heart Apex SRF 1
Adult Heart Apex SRF 2
Adult Heart Apex SRF-input
Adult Heart Apex TBX5 1
Adult Heart Apex TBX5 2
Adult Heart Apex TBX5-input
Adult Heart Apex TEAD1 1
Adult Heart Apex TEAD1 2
Adult Heart Apex TEAD1-input
Fetal Heart Apex MEF2A 1
Fetal Heart Apex MEF2A 2
Fetal Heart Apex MEF2A-input
Fetal Heart Apex MEF2C 1
Fetal Heart Apex MEF2C 2
Fetal Heart Apex MEF2C-input
Fetal Heart Apex NKX25 1
Fetal Heart Apex NKX25 2
Fetal Heart Apex NKX25-input
Fetal Heart Apex SRF 1
Fetal Heart Apex SRF 2
Fetal Heart Apex SRF-input 1
Fetal Heart Apex SRF-input 2
Fetal Heart Apex TBX5 1
Fetal Heart Apex TBX5 2
Fetal Heart Apex TBX5-input 1
Fetal Heart Apex TBX5-input 2
Fetal Heart Apex TEAD1 1
Fetal Heart Apex TEAD1 2
Fetal Heart Apex TEAD1-input 1
Fetal Heart Apex TEAD1-input 2
Fetal Heart-CM ATAC 1
Fetal Heart-CM ATAC 2

Genome browser session
(e.g. UCSC)

https://genome.ucsc.edu/cgi-bin/hgTracks?db=mm9&lastVirtModeType=default&lastVirtModeExtraState=&virtModeType=default&virtMode=0&nonVirtPosition=&position=chr12%3A57803991%2D57810533&hgsid=741360699_YDByfTyaf1JlisQSpA9h5ELj6ZTO

Methodology

Replicates

All bioChIP-seq experiments were done in biologically paired replicates. Fetal bioChIP-seq samples contained approximately 100 dissected embryonic ventricles from pooled litters and this was performed 2X (rep1/2). Adult bioChIP-seq samples contained postnatal day 42 littermates of 2 females/2 males and this was performed 2X (rep1/2).

Sequencing depth

Sample TF rep. IDR peak number reads length sequence type
Adult Heart Apex GATA4 1 19712 7,091,242 50 Single-end
Adult Heart Apex GATA4 2 6,043,489 50 Single-end
Adult Heart Apex GATA4-input 19,707,312 50 Single-end
Adult Heart Apex MEF2A 1 11103 19,165,784 75 Single-end
Adult Heart Apex MEF2A 2 14,523,602 75 Single-end
Adult Heart Apex MEF2A-input 15,050,826 75 Single-end
Adult Heart Apex NKX25 1 20600 10,662,515 75 Single-end
Adult Heart Apex NKX25 2 10,672,783 75 Single-end
Adult Heart Apex NKX25-input 19,685,631 75 Single-end
Adult Heart Apex SRF 1 12485 12,911,177 75 Single-end
Adult Heart Apex SRF 2 24,966,591 75 Single-end
Adult Heart Apex SRF-input 16,276,173 75 Single-end
Adult Heart Apex TBX5 1 6541 17,963,388 75 Single-end
Adult Heart Apex TBX5 2 14,653,611 75 Single-end
Adult Heart Apex TBX5-input 14,806,792 75 Single-end
Adult Heart Apex TEAD1 1 25442 30,520,366 75 Single-end
Adult Heart Apex TEAD1 2 49,532,438 75 Single-end
Adult Heart Apex TEAD1-input 11,045,415 75 Single-end

Fetal Heart Apex GATA4 1 44184 28,234,634 50 Single-end
Fetal Heart Apex GATA4 2 31,535,216 50 Single-end
Fetal Heart Apex GATA4-input 67,462,258 50 Single-end
Fetal Heart Apex MEF2A 1 15933 17,252,802 75 Single-end
Fetal Heart Apex MEF2A 2 29,233,783 75 Single-end
Fetal Heart Apex MEF2A-input 21,427,473 75 Single-end
Fetal Heart Apex MEF2C 1 30178 13,760,038 75 Single-end
Fetal Heart Apex MEF2C 2 14,849,292 75 Single-end
Fetal Heart Apex MEF2C-input 28,363,913 75 Single-end
Fetal Heart Apex NKX25 1 25864 11,879,917 50 Single-end
Fetal Heart Apex NKX25 2 10,387,821 50 Single-end
Fetal Heart Apex NKX25-input 18,076,999 50 Single-end
Fetal Heart Apex SRF 1 18521 11,921,326 75 Single-end
Fetal Heart Apex SRF 2 9,315,410 75 Single-end
Fetal Heart Apex SRF-input 1 9,044,397 75 Single-end
Fetal Heart Apex SRF-input 2 9,315,426 75 Single-end
Fetal Heart Apex TBX5 1 30789 9,097,583 75 Single-end
Fetal Heart Apex TBX5 2 25,525,441 75 Single-end
Fetal Heart Apex TBX5-input 1 16,004,964 75 Single-end
Fetal Heart Apex TBX5-input 2 20,802,737 75 Single-end
Fetal Heart Apex TEAD1 1 45807 26,145,388 75 Single-end
Fetal Heart Apex TEAD1 2 22,285,388 75 Single-end
Fetal Heart Apex TEAD1-input 1 7,462,838 75 Single-end
Fetal Heart Apex TEAD1-input 2 19,063,867 75 Single-end
E12.5 Heart-CM ATAC 1 107586 63,460,950 75 Single-end
E12.5 Heart-CM ATAC 2 58,457,977

Antibodies

bio-tagged chromatin immunoprecipitation (bioChIP) with Invitrogen M280 Streptavidin DynaBeads (cat. 11206D)

Peak calling parameters

BioChIP-Seq: macs2 callpeak -t chip.bam -c input.bam -f BAM -g mm -n chip -p 0.05 --verbose=0
ATAC-seq: macs2 callpeak -f BAM -g mm --keep-dup all --nomodel --nolambda --shift -100 --extsize 200 -B -n
H3K27ac ChIP-seq: macs2 callpeak -t chip.bam -c input.bam -f BAM -g mm -n chip -q 0.01 --verbose=0

Data quality

BioChIP-seq peak numbers are listed below(one per TF biological duplicate pair) Reproducible peaks were identified using IDR (ENCODE metrics) at IDR_THRESHOLD=0.05 between each set of replicate data files. Each IDR peak was represented by the MACS2 summit ± 100 bp of the individual replicate with the greatest peak intensity.
Sample TF rep. IDR peak number
Adult Heart Apex GATA4 1 19712
Adult Heart Apex GATA4 2
Adult Heart Apex GATA4-input
Adult Heart Apex MEF2A 1 11103
Adult Heart Apex MEF2A 2
Adult Heart Apex MEF2A-input
Adult Heart Apex NKX25 1 20600
Adult Heart Apex NKX25 2
Adult Heart Apex NKX25-input
Adult Heart Apex SRF 1 12485
Adult Heart Apex SRF 2
Adult Heart Apex SRF-input
Adult Heart Apex TBX5 1 6541
Adult Heart Apex TBX5 2
Adult Heart Apex TBX5-input
Adult Heart Apex TEAD1 1 25442
Adult Heart Apex TEAD1 2
Adult Heart Apex TEAD1-input
Fetal Heart Apex GATA4 1 44184
Fetal Heart Apex GATA4 2
Fetal Heart Apex GATA4-input
Fetal Heart Apex MEF2A 1 15933
Fetal Heart Apex MEF2A 2
Fetal Heart Apex MEF2A-input
Fetal Heart Apex MEF2C 1 30178
Fetal Heart Apex MEF2C 2
Fetal Heart Apex MEF2C-input
Fetal Heart Apex NKX25 1 25864
Fetal Heart Apex NKX25 2
Fetal Heart Apex NKX25-input
Fetal Heart Apex SRF 2 18521
Fetal Heart Apex SRF 3
Fetal Heart Apex SRF-input 2
Fetal Heart Apex SRF-input 3
Fetal Heart Apex TBX5 1 30789
Fetal Heart Apex TBX5 2
Fetal Heart Apex TBX5-input 1

Fetal Heart Apex TBX5-input 2
Fetal Heart Apex TEAD1 1 45807
Fetal Heart Apex TEAD1 2
Fetal Heart Apex TEAD1-input 1
Fetal Heart Apex TEAD1-input 3
P56 Forebrain H3K27ac 1 33386 (Nord et al., 2013)
P56 Forebrain H3K27ac-input 1
P56 Forebrain H3K27ac 2 (Nord et al., 2013)
P56 Forebrain H3K27ac-input 2
P56 Heart H3K27ac 1 119138 (Nord et al., 2013)
P56 Heart H3K27ac-input 1
P56 Liver H3K27ac 1 103824 (Nord et al., 2013)
P56 Liver H3K27ac-input 1
E11.5 Forebrain H3K27ac 1 8346 (Nord et al., 2013)
E11.5 Forebrain H3K27ac-input 1
E11.5 Forebrain H3K27ac 2 (Nord et al., 2013)
E11.5 Forebrain H3K27ac-input 2
E11.5 Heart H3K27ac 1 37980 (Nord et al., 2013)
E11.5 Heart H3K27ac-input 1
E11.5 Heart H3K27ac 2 (Nord et al., 2013)
E11.5 Heart H3K27ac-input 2
E11.5 Liver H3K27ac 1 33961 (Nord et al., 2013)
E11.5 Liver H3K27ac-input 1
P56 Heart-CM ATAC 1 83430 (Quaife-Ryan et al., 2017)
P56 Heart-CM ATAC 2
P56 Heart-CM ATAC 3
Fetal Heart-CM ATAC 1 107586
Fetal Heart-CM ATAC 2

Software

Bowtie2 was used to align reads to mm9; aligned .bam files were processed using samtools to remove duplicated and blacklisted regions; peaks were called using processed .bam files with MACS2. Deeptools was used to analyze the correlation between bioChIP-seq samples and to generate aggregation plots. To define regions “co-bound” by TFs, the single TF bioChIP-seq peak files were merged using the “mergePeaks” function of Homer, with parameter “-d 300” (merge peaks whose centers are within 300 bp of one another). Motif analysis was performed using Homer. Non-redundant significant motifs in the vertebrate homer database were identified by motif clustering using STAMP followed by manual inspection to select a motif representative of related motifs within each cluster. As an independent, complementary analysis, central enrichment of selected motifs within 1000 bp regions centered on peak summits was evaluated with Centrimo. For composite motif analysis, we generated motif matrices for all 4 possible motif orientations with zero to 8 intervening random bases. These matrices and Homer were used to determine the enrichment of each composite motifs within regions co-occupied by each pairwise combination of TFs. The variance of enrichment across the 32 possible composite motifs was determined by calculating the Fano factor (variance^2/mean). Conservation of regions was analyzed using precalculated phastCons 30-way vertebrate scores. Intersection of the current data with previously reported murine ChIP-seq data (Supplementary Fig. 5) was performed using the R package Intervene. Gene ontology analysis was performed using GREAT and its default rule for associating regions to genes. Permutation analysis was performed using regioneR. Machine learning enhancer prediction used Xgboost package to train an ensemble decision tree model.
