# Supplementary material for: A reference map of murine cardiac transcription factor chromatin occupancy identifies dynamic and conserved enhancers
Source: Nat Commun. 2019 Oct 28;10:4907. doi: 10.1038/s41467-019-12812-3 (PMC6817842; doi:10.1038/s41467-019-12812-3)
Supplement: Supplementary file 10 — Source Data [file 41467_2019_12812_MOESM10_ESM.zip › SourceData_images copy.pdf]

Data Source images

Fig. S1

Genotyping flag-bio alleles

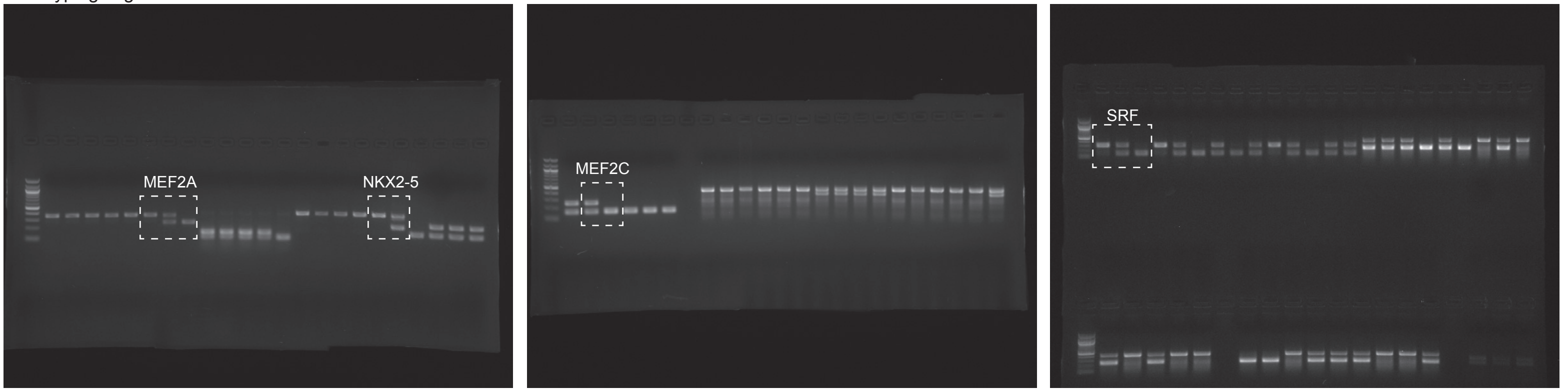

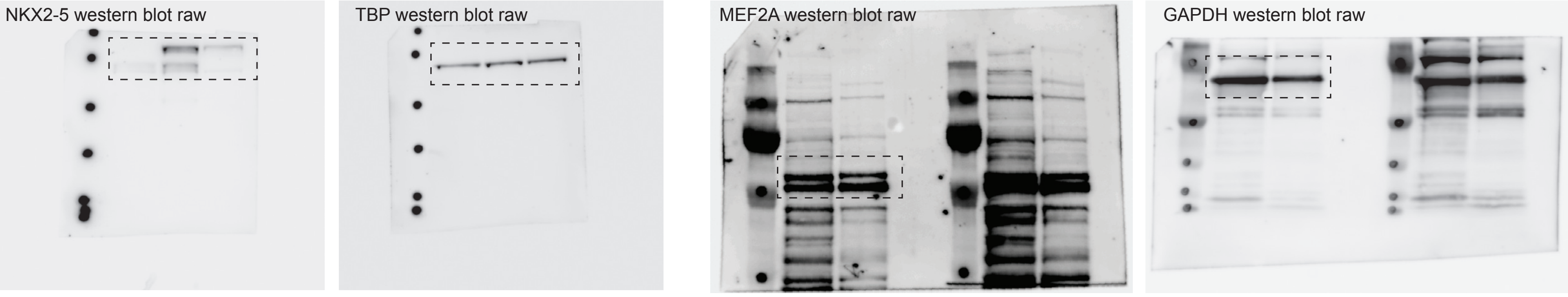

MEF2C protein analysis (WES Protein Simple - high exposure)

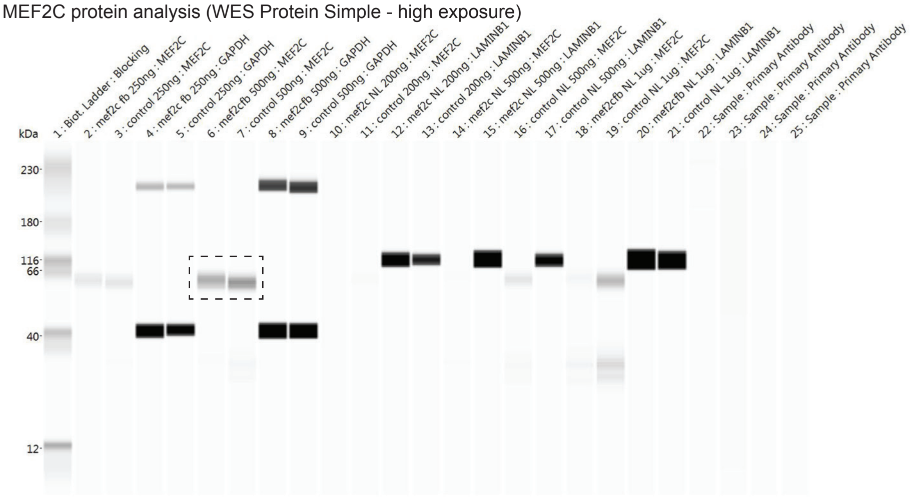

GAPDH protein analysis (WES Protein Simple - low exposure)

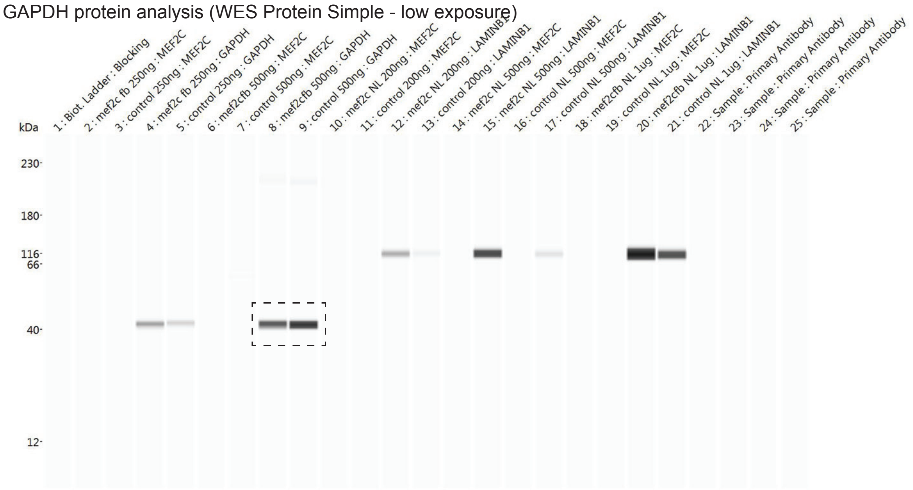

Fig. S9

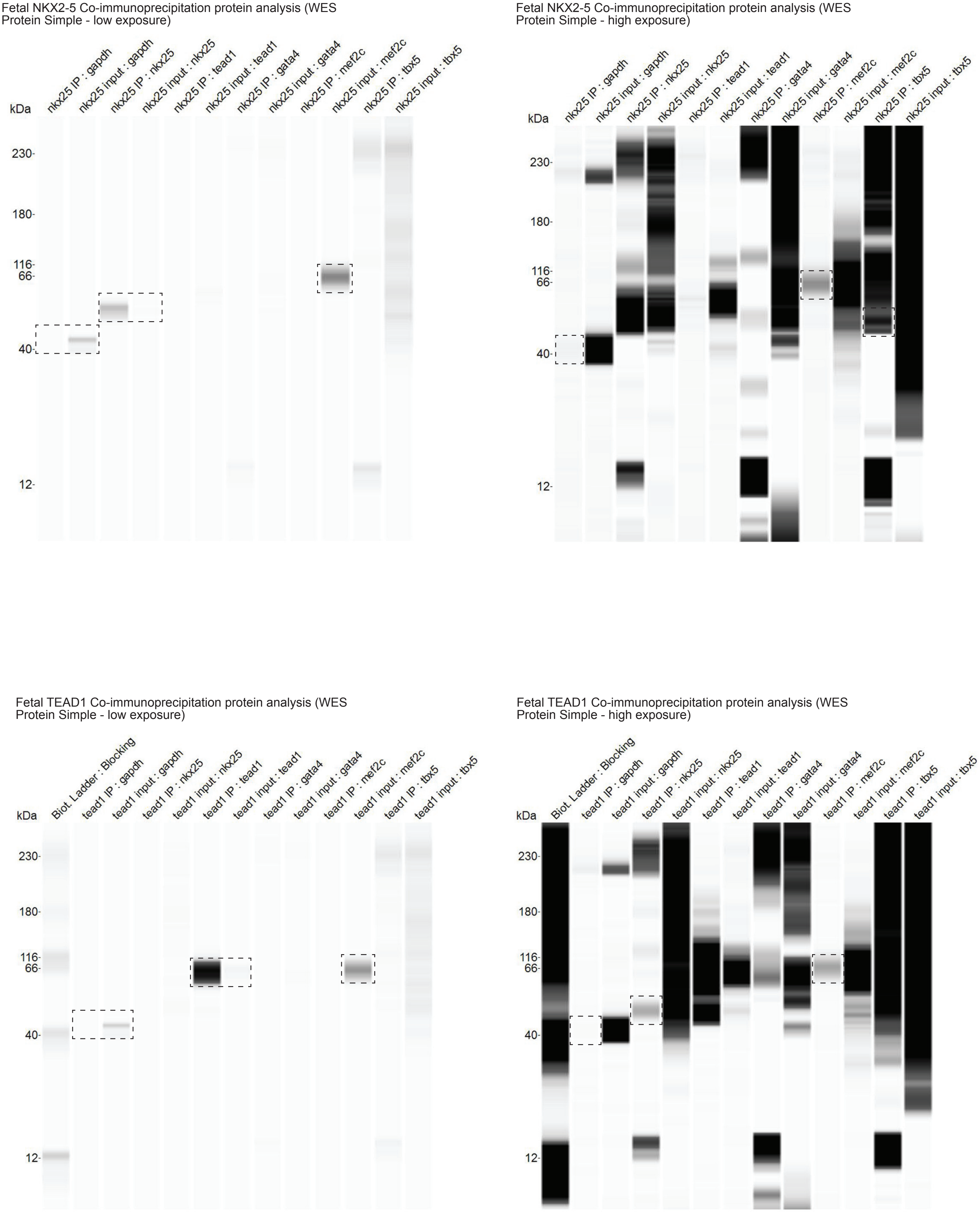
Fetal NKX2-5 Co-immunoprecipitation protein analysis (WES Protein Simple - low exposure)
kDa
nkx25 IP : gapdh
nkx25 input : gapdh
nkx25 IP : nkx25
nkx25 input : nkx25
nkx25 IP : tead1
nkx25 input : tead1
nkx25 IP : gata4
nkx25 input : gata4
nkx25 IP : mef2c
nkx25 input : mef2c
nkx25 IP : tbx5
nkx25 input : tbx5
230
180
116
66
40
12
Fetal NKX2-5 Co-immunoprecipitation protein analysis (WES Protein Simple - high exposure)
Fetal TEAD1 Co-immunoprecipitation protein analysis (WES Protein Simple - low exposure)
Biot. Ladder : Blocking
tead1 IP : gapdh
tead1 input : gapdh
tead1 IP : nkx25
tead1 input : nkx25
tead1 IP : tead1
tead1 input : tead1
tead1 IP : gata4
tead1 input : gata4
tead1 IP : mef2c
tead1 input : mef2c
tead1 IP : tbx5
tead1 input : tbx5
Fetal TEAD1 Co-immunoprecipitation protein analysis (WES Protein Simple - high exposure)
